# *Xrp1* genetically interacts with the ALS-associated *FUS* orthologue *caz* and mediates its toxicity

**DOI:** 10.1083/jcb.201802151

**Published:** 2018-11-05

**Authors:** Moushami Mallik, Marica Catinozzi, Clemens B. Hug, Li Zhang, Marina Wagner, Julia Bussmann, Jonas Bittern, Sina Mersmann, Christian Klämbt, Hannes C.A. Drexler, Martijn A. Huynen, Juan M. Vaquerizas, Erik Storkebaum

**Affiliations:** 1 Molecular Neurogenetics Laboratory, Max Planck Institute for Molecular Biomedicine, Münster, Germany; 2 Faculty of Medicine, University of Münster, Münster, Germany; 3 Department of Molecular Neurobiology, Donders Institute for Brain, Cognition and Behaviour and Radboud University, Nijmegen, Netherlands; 4 Regulatory Genomics, Max Planck Institute for Molecular Biomedicine, Münster, Germany; 5 Bioanalytical Mass Spectrometry Facility, Max Planck Institute for Molecular Biomedicine, Münster, Germany; 6 Centre for Molecular and Biomolecular Informatics, Radboud Institute for Molecular Life Sciences, Radboud University Medical Center, Nijmegen, Netherlands; 7 Institute of Neuro and Behavioural Biology, University of Münster, Münster, Germany

## Abstract

*Cabeza* (*caz*) is the single *Drosophila melanogaster* orthologue of the human FET proteins FUS, TAF15, and EWSR1, which have been implicated in amyotrophic lateral sclerosis (ALS) and frontotemporal dementia. In this study, we identified *Xrp1*, a nuclear chromatin-binding protein, as a key modifier of *caz* mutant phenotypes. Xrp1 expression was strongly up-regulated in *caz* mutants, and *Xrp1* heterozygosity rescued their motor defects and life span. Interestingly, selective neuronal Xrp1 knockdown was sufficient to rescue, and neuronal Xrp1 overexpression phenocopied *caz* mutant phenotypes. The *caz/Xrp1* genetic interaction depended on the functionality of the AT-hook DNA-binding domain in Xrp1, and the majority of Xrp1-interacting proteins are involved in gene expression regulation. Consistently, *caz* mutants displayed gene expression dysregulation, which was mitigated by *Xrp1* heterozygosity. Finally, *Xrp1* knockdown substantially rescued the motor deficits and life span of flies expressing ALS mutant FUS in motor neurons, implicating gene expression dysregulation in ALS-FUS pathogenesis.

## Abstract (precis)

Mallik et al. identify Xrp1 as a nuclear chromatin-binding protein involved in gene expression regulation that mediates phenotypes induced by loss of function of *cabeza* (*caz*), the *Drosophila melanogaster* orthologue of amyotrophic lateral sclerosis (ALS) and frontotemporal dementia (FTD) protein FUS. Knockdown of Xrp1 in motor neurons rescues phenotypes induced by ALS-mutant FUS.

## Introduction

Amyotrophic lateral sclerosis (ALS) is an adult-onset neurodegenerative disorder characterized by motor neuron loss, leading to progressive muscle weakness and ultimately complete paralysis and death (Taylor et al., 2016). Mutations in several genes encoding RNA-binding proteins (RBPs) cause familial ALS (FALS), including TDP-43 (Gitcho et al., 2008; Kabashi et al., 2008; Sreedharan et al., 2008), FUS (Kwiatkowski et al., 2009; Vance et al., 2009), TAF15 (Couthouis et al., 2011), EWSR1 (Couthouis et al., 2012), hnRNPA1 and hnRNPA2B1 (Kim et al., 2013b), and matrin-3 (Johnson et al., 2014). Furthermore, TDP-43–positive inclusions are found in most sporadic ALS patients (Neumann et al., 2006; Taylor et al., 2016), and inclusions containing either TDP-43 or FUS are a pathological hallmark in ∼45% and ∼10% of patients with frontotemporal dementia (FTD), respectively (Ling et al., 2013). These findings implicated defects in RNA biogenesis in ALS and FTD pathogenesis.

Of the ALS-associated RBPs, FUS, EWSR1, and TAF15 (FET) proteins are highly homologous proteins that constitute the FET family (Schwartz et al., 2015). The FET proteins are DNA-binding proteins and RBPs involved in gene expression regulation, including transcription, mRNA splicing, and mRNA subcellular localization (Schwartz et al., 2015). Heterozygous mutations in *FUS* account for ∼5% of FALS (Ling et al., 2013), while mutations in TAF15 and EWSR1 are rare (Couthouis et al., 2011, 2012). Most ALS-associated mutations cluster in the nuclear localization signal of FUS, resulting in a shift from a predominantly nuclear to a more cytoplasmic localization, formation of cytoplasmic aggregates, and reduced nuclear FUS levels (Da Cruz and Cleveland, 2011). This suggests that loss of nuclear FUS function may contribute to ALS pathogenesis, although evidence from ALS-FUS mouse models indicates that ALS-FUS mutations also result in a novel toxic function that triggers motor neuron degeneration (Scekic-Zahirovic et al., 2016, 2017; Sharma et al., 2016). Moreover, in FTD with FUS pathology (FTLD-FUS), the three FET proteins are found in pathogenic inclusions, with reduced levels or complete loss of nuclear FET proteins in inclusion-bearing cells, indicating that loss of nuclear FET function may contribute to FTLD-FUS (Neumann et al., 2011; Davidson et al., 2013).

The *Drosophila melanogaster* gene *cabeza* (*caz*) encodes the single fly orthologue of the three human FET proteins. Accordingly, Caz is a predominantly nuclear RBP that contains the functional domains of the human FET proteins (Schwartz et al., 2015). When expressed in mammalian cells, Caz elicits down-regulation of FUS protein levels (Immanuel et al., 1995), while FUS expression in *Drosophila* rescues *caz* mutant phenotypes (Wang et al., 2011), indicating functional homology. We previously generated *caz* mutant animals, which exhibit pupal lethality because adult flies fail to eclose due to motor deficits (Frickenhaus et al., 2015). In this study, we performed a genetic screen to gain insight into the molecular mechanisms underlying *caz* mutant phenotypes. Exhaustive screening of ∼80% of the *Drosophila* genome identified *Xrp1* as the only gene for which heterozygosity could rescue *caz* mutant phenotypes. *Xrp1* encodes a protein containing an AT-hook DNA-binding domain often found in proteins involved in chromatin remodeling, transcriptional regulation, and DNA repair (Reeves, 2010). *Xrp1* expression was increased in *caz* mutants, and neuron-selective knockdown of *Xrp1* was sufficient to rescue *caz* mutant phenotypes. Importantly, the DNA-binding capacity of the AT-hook domain of Xrp1 was required to mediate *caz* mutant phenotypes, and *caz* mutants displayed substantial gene expression dysregulation, which was significantly mitigated by heterozygosity for *Xrp1*. Finally, Xrp1 knockdown in motor neurons rescued phenotypes induced by ALS mutant FUS expression, underscoring the potential relevance of our findings for human disease. Together, we propose that *caz* mutant phenotypes are mediated by up-regulation of *Xrp1*, leading to gene expression dysregulation and neuronal dysfunction.

## Results

### A genetic screen to identify suppressors of *caz* mutant phenotypes

**Figure 1.**
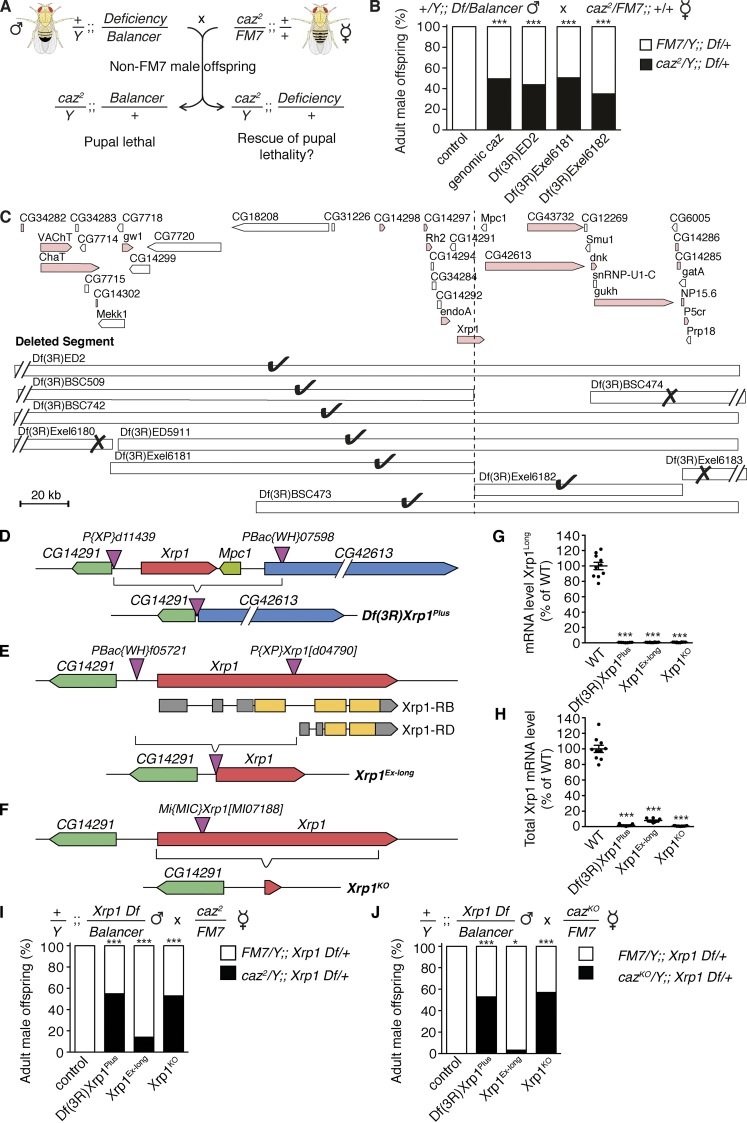
**Heterozygosity for *Xrp1* rescues *caz* mutant pupal lethality. (A)** Screening strategy to identify chromosomal deficiencies that rescue *caz^2^* pupal lethality. **(B)** Frequency of adult male offspring from the indicated cross that is heterozygous for a genomic *caz* transgene or the indicated deficiencies. *n* > 128 per genotype. ***, P < 0.0001; χ^2^ test. **(C)** Genomic region uncovered by *Df(3R)ED2*. Pink indicates genes in the plus orientation; white indicates genes in the minus orientation. The different smaller deficiencies within this region which were tested for rescue of *caz^2^* pupal lethality are shown. Check marks indicate deficiencies that rescue; X marks indicate deficiencies that do not rescue. **(D–F)** *Xrp1* genomic locus showing the insertion sites of the transposable elements used to generate *Xrp1* mutant alleles. In the *Df(3R)Xrp1^Plus^* allele (D), *Xrp1*, *Mpc1*, and the 5′ end of *CG42613* are deleted. In the *Xrp1^Ex-long^* allele (E), the 5′ half of *Xrp1* is deleted, predicted to abolish expression of the Xrp1^Long^ isoform. The Xrp1^Short^ isoform, encoded by Xrp1-RD, may still be expressed. In the *Xrp1^KO^* allele (F), the *Xrp1* coding region is precisely deleted. **(G and H)** Xrp1 transcript levels in *Xrp1* mutant lines relative to WT controls (100%) determined by qPCR using primers either selectively detecting Xrp1^Long^ transcripts (G) or detecting all Xrp1 transcripts (H). *n* = 10. ***, P < 0.0001; one-way ANOVA. Mean ± SEM. **(I and J)** Frequency of adult male offspring from the indicated crosses that is heterozygous for the indicated *Xrp1* allele. *n* > 87 per genotype. *, P < 0.05; ***, P < 0.0001; χ^2^ test.

We previously generated two independent *caz* null alleles: (1) *caz^2^*, an imprecise excision allele, and (2) *caz^KO^*, generated by homologous recombination (Frickenhaus et al., 2015). *Caz* mutants die during the pupal stage due to motor incapability resulting in pharate adults failing to eclose from the pupal case. This phenotype was used to perform a dominant suppressor screen whereby males carrying chromosomal deficiencies were crossed to *caz^2^* heterozygous females. Since *caz* is on the X chromosome, this approach allowed us to screen for genes on the second and third chromosomes for which hemizygosity would rescue the pupal lethality of *caz^2^* males (Fig. 1 A). This screen yielded only a single deficiency that rescued *caz^2^* pupal lethality, *Df(3R)ED2* (Fig. 1 B).

Fine mapping using smaller overlapping deficiencies reduced the number of candidate genes to 11 (Fig. 1 C). As *Df(3R)Exel6181* and *Df(3R)Exel6182* are neighboring but nonoverlapping deficiencies that have a common break point in *Xrp1*, the fact that heterozygosity for either of these deficiencies rescued *caz^2^* pupal lethality suggested that heterozygous loss of *Xrp1* may mediate the rescue (Fig. 1, B and C).

### Heterozygosity for *Xrp1* rescues *caz* mutant pupal lethality

*Xrp1* is predicted to encode seven alternative transcripts (Fig. S1 A), four of which can be translated into a 668-aa “long” isoform (Xrp1^Long^) and the remaining three into a 406-aa “short” isoform (Xrp1^Short^). We generated two *Xrp1* deletion alleles: *Df(3R)Xrp1^Plus^* is a ∼25-kb deletion of *Xrp1*, *Mpc1*, and part of *CG42613* (Fig. 1 D), whereas *Xrp1^Ex-long^* selectively deletes two thirds of *Xrp1*, expected to abolish expression of all Xrp1^Long^ transcripts, but possibly leaving expression of a Xrp1^Short^ transcript (Xrp1-RD) intact (Fig. 1 E). In addition, we used in vivo homologous recombination to generate the *Xrp1^KO^* allele, in which the entire *Xrp1* coding region is precisely deleted (Figs. 1 F and S1 C).

All three *Xrp1* mutant alleles were homozygous viable, and quantitative PCR (qPCR) using primers that selectively detect Xrp1^Long^ revealed loss of Xrp1^Long^ transcript in homozygous *Df(3R)Xrp1^Plus^*, *Xrp1^Ex-long^*, and *Xrp1^KO^* animals (Fig. 1 G). qPCR using primers detecting all Xrp1 isoforms revealed loss of Xrp1 transcript in *Df(3R)Xrp1^Plus^* and *Xrp1^KO^* flies, whereas in *Xrp1^Ex-long^* flies, some residual Xrp1 transcript could be detected (∼8% of WT levels), presumably reflecting expression of the short Xrp1-RD mRNA isoform (Fig. 1 H).

Crossing males heterozygous for either *Df(3R)Xrp1^Plus^*, *Xrp1^Ex-long^*, or *Xrp1^KO^* to *caz^2^/FM7* or *caz^KO^/FM7* females revealed that heterozygosity for *Df(3R)Xrp1^Plus^* or *Xrp1^KO^* rescued *caz* mutant pupal lethality to a similar extent as *Xrp1* deficiencies and a genomic *caz* transgene, independent of the *caz* null allele used (Fig. 1, I and J). Heterozygosity for *Xrp1^Ex-long^* only partially rescued, presumably due to residual Xrp1 expression (Fig. 1, I and J). Furthermore, the mild rough eye phenotype previously reported in *caz* mutant adult escaper flies (Wang et al., 2011) was rescued by *Xrp1* heterozygosity. While a genetic interaction was previously reported between *caz* and *TBPH*, the *Drosophila* homologue of *TARDBP*, encoding TDP-43 (Wang et al., 2011), *Xrp1* heterozygosity could not rescue the adult eclosion defect of *TBPH* mutants (Fig. S2). Thus, loss of 50% of *Xrp1* gene dosage is sufficient to rescue pupal lethality induced by loss of *caz* but not *TBPH* function.

### *Xrp1* heterozygosity rescues motor deficits and life span of *caz* mutant flies

**Figure 2.**
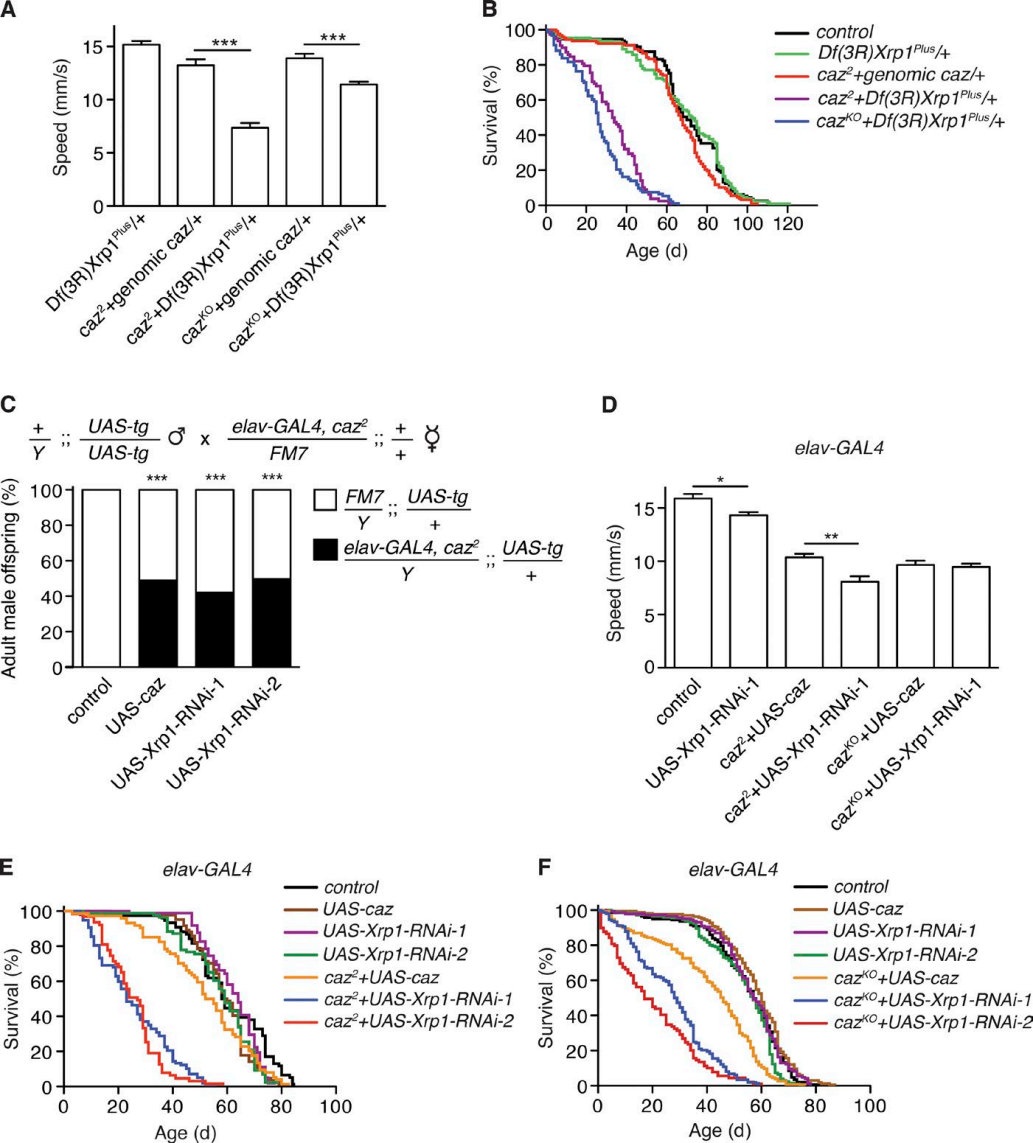
**Rescue of *caz* mutant phenotypes by *Xrp1* heterozygosity or by selective knockdown of Xrp1 in neurons. (A)** Average climbing speed in an automated negative geotaxis assay of heterozygous *Df(3R)Xrp1^Plus^* male flies (control) and *caz* mutant males rescued by genomic *caz* or heterozygosity for *Xrp1*. *n* > 100 per genotype. ***, P < 0.0005; Mann-Whitney test. Mean ± SEM. **(B)** Life span of WT (control), *Df(3R)Xrp1^Plus^* heterozygous, and *caz* mutant male flies rescued by genomic *caz* or heterozygosity for *Xrp1*. *n* = 78–127 per genotype. **(C)** Frequency of adult male offspring from the indicated cross that carries UAS-caz, UAS-Xrp1-RNAi, or no UAS transgene (control). *n* > 176 per genotype. ***, P < 0.0001; χ^2^ test. **(D)** Average climbing speed of adult male control (driver-only) flies, flies with neuronal (*elav-GAL4*) Xrp1 knockdown, and *caz* mutants rescued by neuronal Caz or neuronal Xrp1 knockdown. *n* > 100 per genotype. *, P < 0.05; **, P < 0.01; Mann-Whitney test. Mean ± SEM. **(E and F)** Life span of male flies selectively expressing caz or Xrp1-RNAi in neurons either in a WT, *caz^2^* (E), or *caz^KO^* (F) background. *n* = 76–164 per genotype.

As *caz* mutant flies display motor defects (Wang et al., 2011; Frickenhaus et al., 2015), we evaluated motor performance of *caz* mutant *Xrp1* heterozygous flies using an automated negative geotaxis climbing assay (Niehues et al., 2015). While we failed to obtain *caz* mutant adult escapers, *Xrp1* heterozygous *caz* mutant flies managed to climb the wall of a test vial, although their climbing speed was still significantly reduced as compared with control animals (Fig. 2 A). Analysis of larval locomotion revealed that *caz* mutant third instar larvae display a significantly reduced crawling speed, which was fully rescued by heterozygosity for *Xrp1* (Fig. S3 A).

Evaluation of life span revealed that *Xrp1* heterozygosity resulted in a substantial rescue of life span as compared with pupal lethality of *caz^2^* and *caz^KO^* flies, although the life span of rescued *caz* mutants was still shorter than control flies (Fig. 2 B). Thus, heterozygosity for *Xrp1* partially but substantially rescues *caz* mutant motor performance and life span.

### Selective knockdown of *Xrp1* in neurons rescues *caz* mutant phenotypes

Selective reintroduction of Caz in neurons was shown to rescue *caz* mutant phenotypes (Wang et al., 2011; Frickenhaus et al., 2015), while selective inactivation of *caz* in neurons resulted in severe motor deficits and reduced life span (Frickenhaus et al., 2015), indicating that loss of *caz* function in neurons is both necessary and sufficient to induce *caz* mutant phenotypes. We therefore evaluated whether selective knockdown of *Xrp1* in neurons was sufficient to rescue *caz* mutant phenotypes despite the fact that the FlyAtlas and modENCODE databases report *Xrp1* expression in all tissues throughout development and adult life. Two independent transgenic Xrp1-RNAi lines revealed that selective knockdown of *Xrp1* in neurons (*elav-GAL4*) fully rescued *caz* mutant pupal lethality (Fig. 2 C) and partially rescued *caz* mutant motor performance for *caz^KO^* even to a similar extent as neuronal Caz reintroduction (Fig. 2 D). Importantly, selective knockdown of *Xrp1* in motor neurons (*D42-GAL4*) was sufficient to fully rescue the reduced crawling speed of *caz* mutant larvae (Fig. S3 B), indicating that the larval locomotion deficit is attributable to dysfunction of motor neurons. Finally, the median life span of *caz* mutant flies with neuronal Xrp1 knockdown ranged from 30% to 50% of the life span of their respective controls (Fig. 2, E and F), comparable with the life span of *Xrp1* heterozygous *caz* mutants. To evaluate the level of knockdown induced by the two Xrp1-RNAi lines, qPCR revealed residual Xrp1 transcript levels in the central nervous system (CNS) of *actin5C-GAL4*>UAS-Xrp1-RNAi third instar larvae of ∼7% and ∼11% of control levels for UAS-Xrp1-RNAi-1 and UAS-Xrp1-RNAi-2, respectively (Fig. S1 B). Furthermore, selective Xrp1 knockdown in glial cells did not rescue *caz* mutant pupal lethality (Fig. S4 A). Together, these findings demonstrate that selective down-regulation of *Xrp1* in neurons is sufficient to rescue *caz* mutant phenotypes.

### Increased *Xrp1* expression mediates *caz* mutant phenotypes

**Figure 3.**
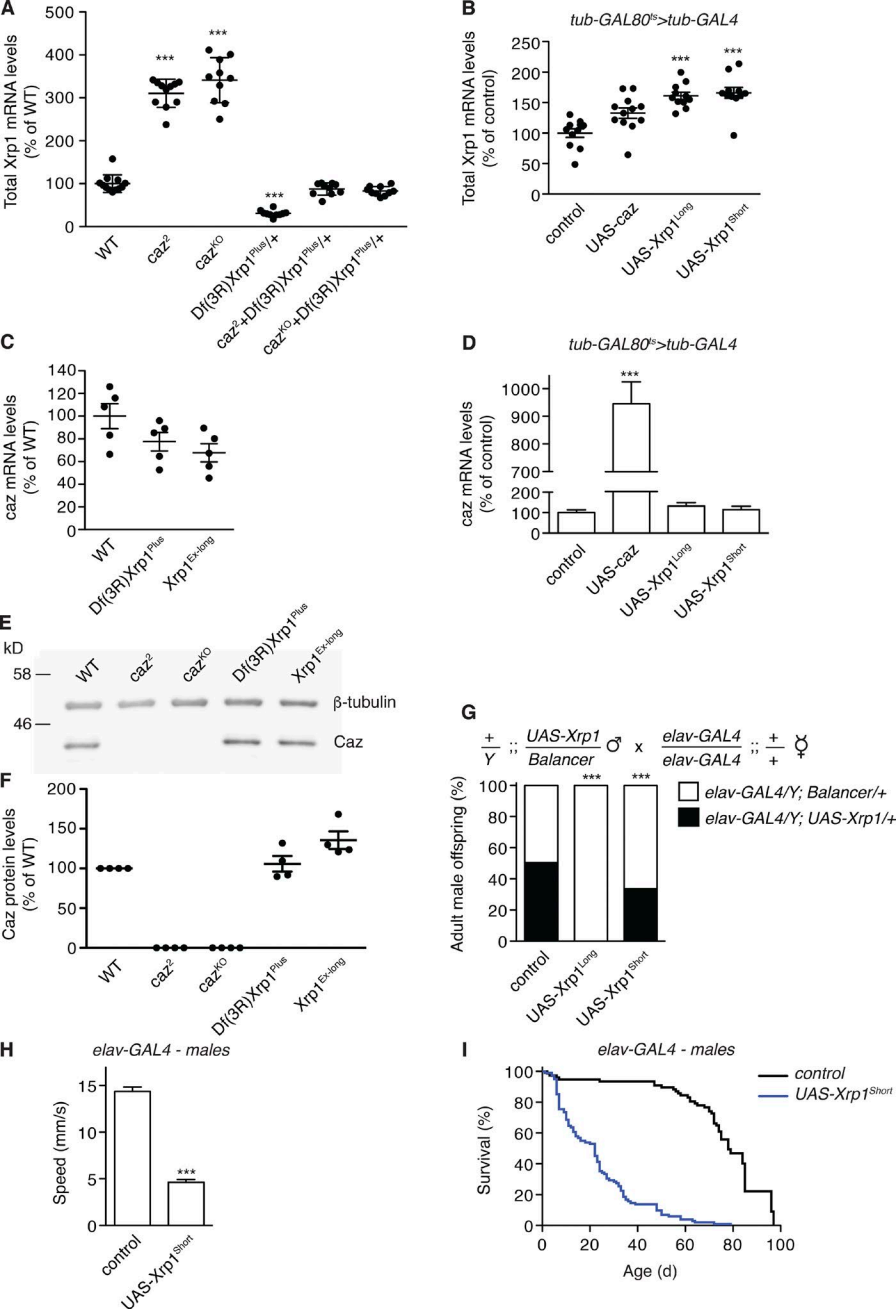
**Xrp1 expression is up-regulated in *caz* mutants, and selective neuronal Xrp1 overexpression phenocopies *caz* mutant phenotypes. (A)** Xrp1 transcript levels as determined by qPCR on CNS of WT, *caz* mutant, *Xrp1* heterozygous, and *caz* mutant *Xrp1* heterozygous larvae. *n* = 10. **(B)** Xrp1 transcript levels in heads of adult male flies that ubiquitously (*tub-GAL4*) overexpress Caz, Xrp1^Long^, Xrp1^Short^, or no transgene (control) from the adult stage onwards. *n* = 10. **(C)** Caz transcript levels in larval CNS of WT and two *Xrp1* mutants. *n* = 10. **(D)** Caz transcript levels in heads of adult male flies that ubiquitously overexpress caz, Xrp1^Long^, Xrp1^Short^, or no transgene (control) from the adult stage onwards. *n* = 10. ***, P < 0.0001; one-way ANOVA. **(E)** Representative Western blot to evaluate Caz protein levels in larval CNS from WT, *caz* mutants, and *Xrp1* mutants. β-tubulin was used as loading control. **(F)** Quantification of Caz protein levels relative to β-tubulin. *n* = 5. P = NS; one-way ANOVA. **(G)** Frequency of adult male offspring from the indicated cross. *n* > 111 per genotype. ***, P < 0.0005; χ^2^ test. **(H)** Average climbing speed of adult male flies selectively overexpressing Xrp1^Short^ in neurons (*elav-GAL4*) as compared with driver-only controls. *n* > 100 per genotype. ***, P < 10^−9^; Mann-Whitney test. **(I)** Life span of male flies selectively overexpressing Xrp1^Short^ in neurons (*elav-GAL4*) as compared with driver-only controls. *n* = 77–102. All graphs display mean ± SEM.

Our finding that reducing *Xrp1* expression rescues *caz* mutant phenotypes raised the possibility that these phenotypes are caused by up-regulation of *Xrp1* expression, with particularly deleterious effects in neurons. Consistent with this hypothesis, qPCR revealed that Xrp1 mRNA levels are increased three- to fourfold in both CNS and body wall of *caz* mutants (Figs. 3 A and S4, B and C). Remarkably, in *caz* mutant *Xrp1* heterozygotes, Xrp1 transcript levels were not significantly different from controls (Fig. 3 A). Thus, phenotypic rescue of *caz* mutants by *Xrp1* heterozygosity is associated with normalization of Xrp1 expression levels. Vice versa, ubiquitous *caz* overexpression from the adult stage onwards did not reduce Xrp1 mRNA levels (Fig. 3 B). To evaluate whether *Xrp1* gene dosage modifies *caz* expression levels, qPCR and Western blotting was performed on *Xrp1* mutants and transgenic flies ubiquitously overexpressing Xrp1^Long^ or Xrp1^Short^. These analyses revealed that Caz levels are not significantly changed in *Xrp1* mutants (Fig. 3, C, E, and F) or upon Xrp1 overexpression (Fig. 3 D). Thus, loss of *caz* increases *Xrp1* expression, but Caz overexpression does not down-regulate Xrp1, and alteration of Xrp1 levels has no effect on *caz* expression.

To further test the hypothesis that increased Xrp1 expression in neurons is a key mediator of *caz* mutant phenotypes, we evaluated the effect of selective Xrp1 overexpression in neurons of otherwise WT flies. Neuronal overexpression of either Xrp1^Long^ or Xrp1^Short^ induced developmental lethality, with a fraction of adult escapers emerging (Figs. 3 G and S4 D). These adult escapers displayed substantial motor performance deficits (Figs. 3 H and S4 E) and a significantly shortened life span (Figs. 3 I and S4 F). Thus, neuronal Xrp1 overexpression phenocopies *caz* mutant phenotypes. Together with the findings that *Xrp1* heterozygosity or Xrp1 neuronal knockdown rescue *caz* mutant phenotypes, these results indicate that increased neuronal Xrp1 levels mediate *caz* mutant phenotypes.

### Xrp1 is a nuclear protein that binds chromatin

The Xrp1 protein is predicted to contain two conserved DNA-binding domains in its C terminus: (1) an AT-hook motif consisting of nine amino acids centered on the invariant tripeptide glycine-arginine-proline (Reeves, 2010) and (2) a basic-region leucine zipper (bZIP) motif found in the bZIP family of transcription factors, which typically consists of a basic region of ∼20 aa that mediates sequence-specific DNA binding along with a leucine zipper, a sequence of 40–60 hydrophobic amino acids in which leucine occurs every seventh residue, which mediates dimerization (Vinson et al., 2002). Despite the fact that Xrp1 was reported to heterodimerize with the bZIP protein Irbp18 (Francis et al., 2016), coimmunoprecipitation experiments on extracts of *Drosophila* S2 cells cotransfected with N-terminal HA-tagged and Flag-tagged variants of either Xrp1^Long^ or Xrp1^Short^ indicated that Xrp1 does not homodimerize (Fig. S4, G and H).

As Ensembl and NCBI Blastp searches failed to identify a human Xrp1 orthologue, we used HHpred (Zimmermann et al., 2018), the most sensitive homology detection tool, to identify human Xrp1 homologues. This yielded human homologues for the C-terminal ∼150 aa of Xrp1 that contain the conserved DNA-binding domains (Table S1). Although these human proteins all contain a bZIP domain, they do not appear to be Xrp1 orthologues, as reciprocal searches against *Drosophila* proteins using HHpred with the “best hits” from human, a reliable method to detect orthologues whose sequence homology is not apparent with pairwise searches (Szklarczyk et al., 2012), did not uncover Xrp1. Furthermore, none of the human Xrp1 homologues contained an AT-hook motif, and in fact, none of the bZIP proteins in the SMART database (Letunic and Bork, 2018) contained an AT-hook motif.

**Figure 4.**
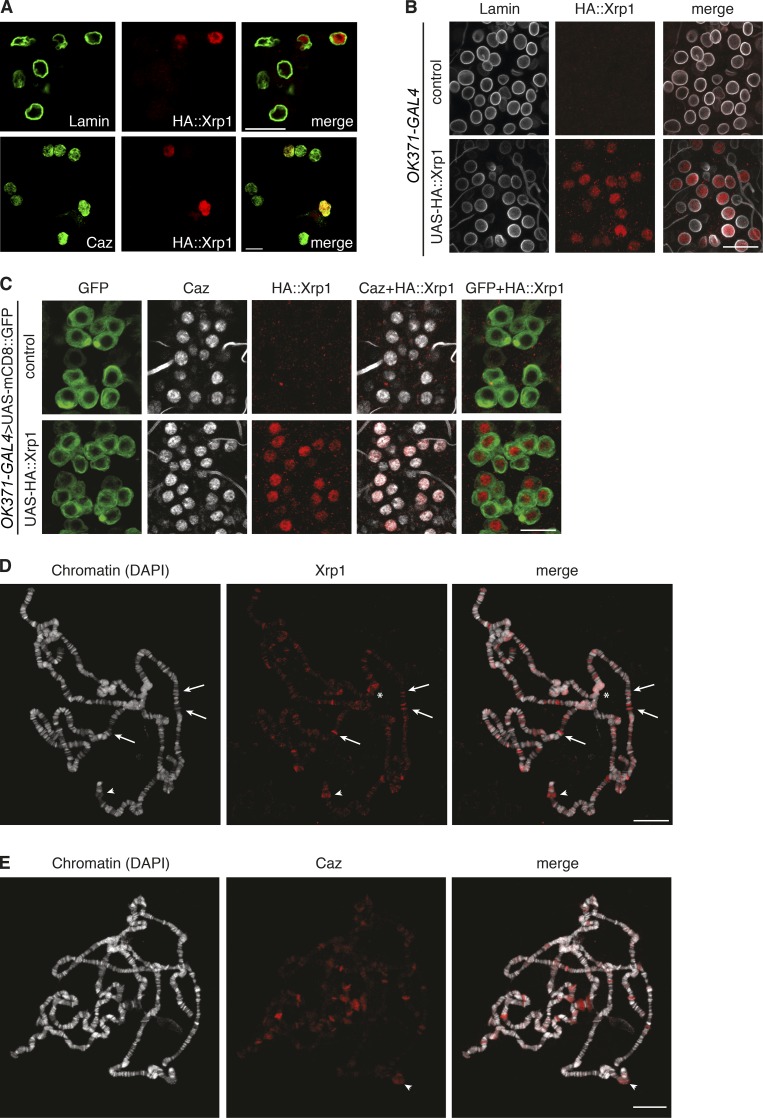
**Xrp1 is a nuclear protein that binds chromatin. (A)** Immunostaining of *Drosophila* S2R^+^ cells expressing N-terminal HA-tagged Xrp1^Short^ for lamin (labels the nuclear membrane), and the HA tag revealed Xrp1 localization to the nucleus (top). Immunostaining for Caz and HA::Xrp1 showed colocalization (bottom). **(B)** HA-tagged Xrp1 was selectively expressed in larval motor neurons (*OK371-GAL4*). Immunostaining for lamin and HA::Xrp1 revealed nuclear localization. Control animals are driver only. **(C)** HA-tagged Xrp1 was coexpressed with membrane-bound GFP in larval motor neurons. Immunolabeling for Caz and HA::Xrp1 showed colocalization of Caz and Xrp1. Control animals do not express HA::Xrp1. **(D and E)** Immunostaining of Xrp1 (D) or Caz (E) on polytene chromosomes. Chromatin was counterstained with DAPI. Xrp1 prominently localizes to euchromatic bands (arrows indicate examples), to puffs (arrowheads), and to centromeric β-heterochromatin (asterisks). Caz prominently localizes to puffs. Overall, Caz and Xrp1 display a distinct binding pattern, although there is some overlap, e.g., the puff region indicated by an arrowheads in D and E. Bars: 10 µm (A); 20 µm (B–E).

Consistent with the presence of two putative DNA-binding domains and its reported roles in protection against genotoxic stress and DNA repair (Brodsky et al., 2004; Akdemir et al., 2007; Francis et al., 2016), subcellular localization experiments revealed that Xrp1 is localized to the nucleus, where it colocalizes with Caz, both in *Drosophila* S2R^+^ cells and in motor neurons in vivo (Fig. 4, A–C). To evaluate whether Xrp1 binds chromatin, immunostaining for Xrp1 was performed on polytene chromosomes from larval salivary glands. Xrp1 was found to preferentially localize to euchromatic bands and to “puffs,” enlarged regions which indicate sites of active transcription (Fig. 4 D), suggesting a possible involvement of Xrp1 in regulation of gene expression. Furthermore, Xrp1 also localized to centromeric β-heterochromatin (Fig. 4 D). Caz prominently localized to puffs on polytene chromosomes (Fig. 4 E), and overall, Caz and Xrp1 displayed a distinct binding pattern with some overlap (e.g., arrowhead in Fig. 4, D and E).

### Xrp1-interacting proteins suggest a role in gene expression regulation

**Figure 5.**
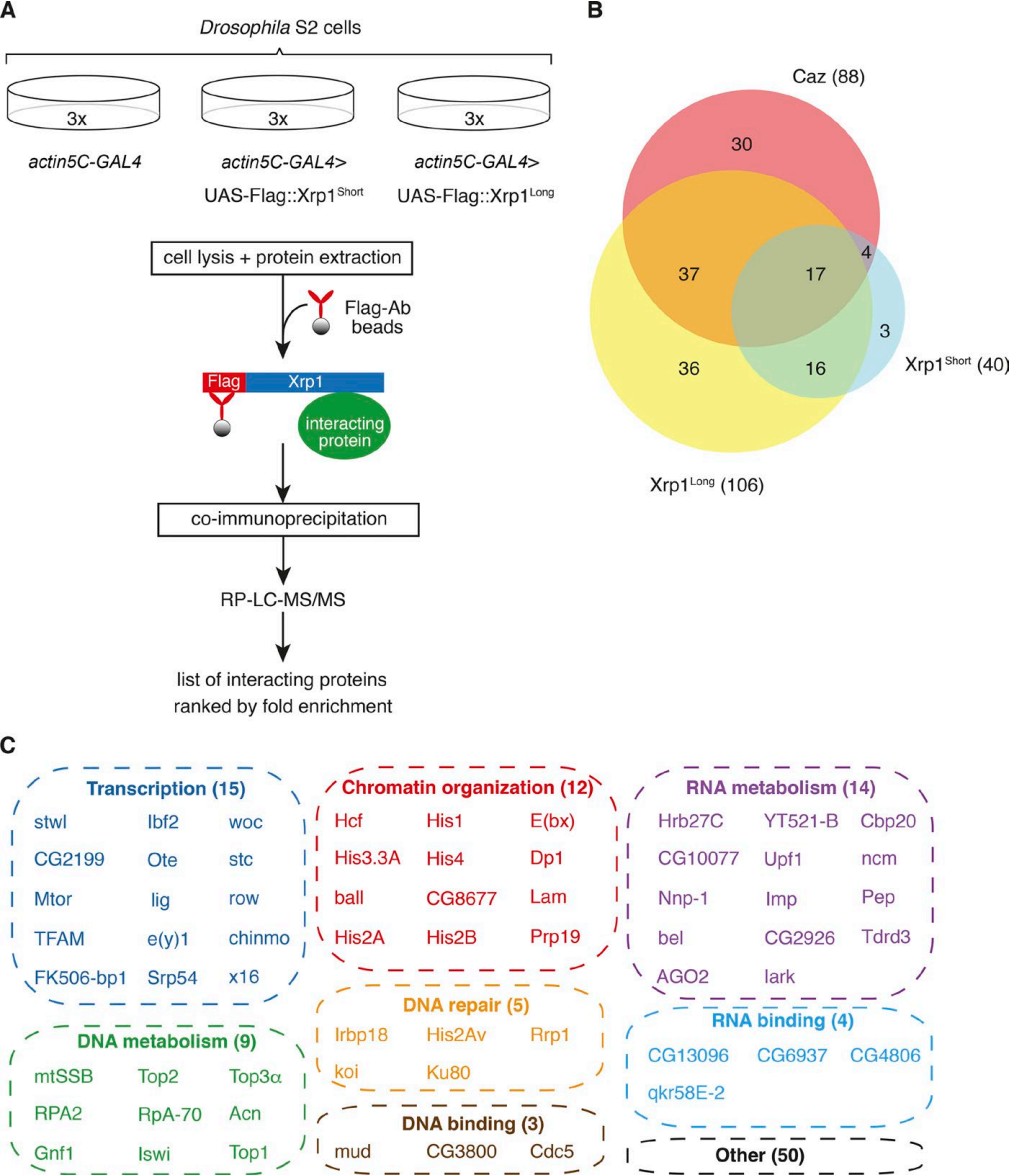
**Identification of Xrp1- and Caz-interacting proteins. (A)** Approach used to identify Xrp1-interacting proteins. N-terminal Flag-tagged Xrp1 was expressed in *Drosophila* S2 cells. Coimmunoprecipitation followed by quantitative MS was used to identify Xrp1-interacting proteins, defined as proteins significantly enriched in Flag::Xrp1-expressing cells as compared with control cells. **(B)** Venn diagrams illustrating the substantial overlap between Xrp1^Long^-, Xrp1^Short^-, and Caz-interacting proteins. **(C)** The majority (55.4%) of Xrp1-interacting proteins are involved in gene expression regulation or DNA/RNA metabolism, including regulation of transcription, chromatin organization, DNA metabolism, DNA repair, RNA metabolism, and DNA- or RNA-binding proteins.

To obtain a comprehensive overview of the molecular processes in which Xrp1 may be involved, we immunoprecipitated N-terminal Flag-tagged Xrp1 (either short or long isoform) from *Drosophila* S2 cells and identified interacting proteins by mass spectrometry (MS; Fig. 5 A). 106 Xrp1^long^-interacting proteins and 40 Xrp1^short^-interacting proteins were identified (Tables S2 and S3). The substantially higher number of Xrp1^long^-interacting proteins is likely attributable to its 262 additional N-terminal amino acids. Importantly, of the 40 Xrp1^short^-interacting proteins, 33 were also identified as Xrp1^long^-interacting proteins (Fig. 5 B). In addition, consistent with heterodimer formation between Xrp1 and Irbp18 (Francis et al., 2016), Irbp18 was identified as an Xrp1-interacting protein (Fig. 5 C and Table S2). Remarkably, out of a total of 112 Xrp1-interacting proteins, 62 (55.4%) are involved in gene expression regulation or DNA/RNA metabolism, including regulation of transcription (15), chromatin organization (12), DNA metabolism (9), DNA repair (5), RNA metabolism (14), and DNA-binding proteins (3) and RBPs (4; Fig. 5 C).

Following a similar experimental approach, we immunoprecipitated endogenous Caz from S2 cells and identified 88 Caz-interacting proteins (Table S4). Remarkably, 58 of these proteins (65.9%) were also identified as Xrp1-interacting proteins (Fig. 5 B), suggesting that Caz and Xrp1 are involved in similar molecular processes and/or commonly reside in protein complexes. The latter possibility is unlikely, as Caz did not coimmunoprecipitate with Xrp1 (Fig. S4 I).

### The *caz-Xrp1* genetic interaction depends on the functionality of the AT-hook domain of Xrp1

We next wanted to evaluate whether the rescue of *caz* mutant phenotypes by *Xrp1* heterozygosity depends on the functionality of the AT-hook DNA-binding domain of Xrp1. We therefore evaluated whether a mild increase of Xrp1 levels in “rescued” *Xrp1* heterozygous *caz* mutant flies would revert the rescue and result in pupal lethality, and if so, whether expression of Xrp1 with a subtle mutation in the AT-hook motif that precludes DNA binding would still revert the rescue.

**Figure 6.**
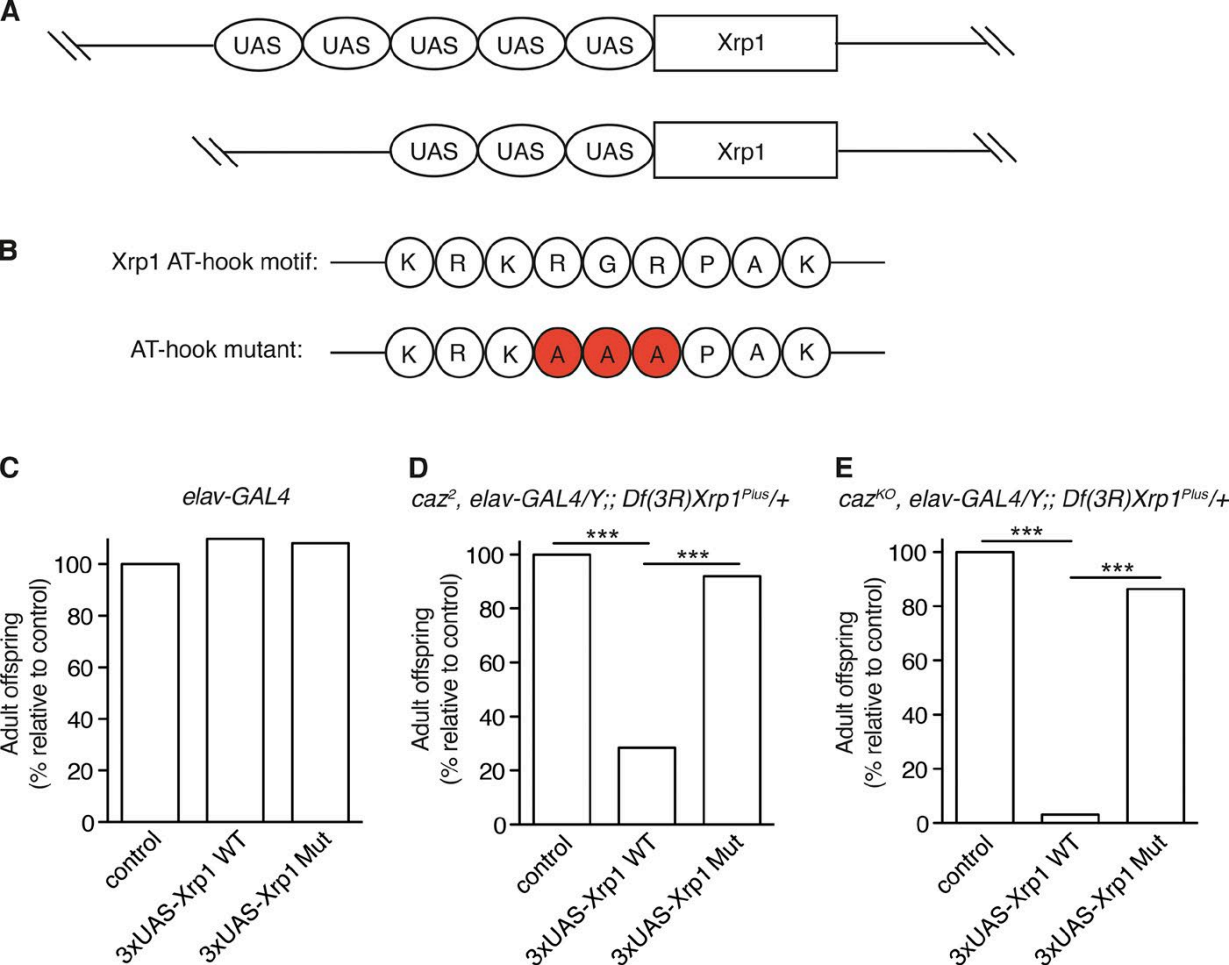
**Functionality of the AT-hook DNA-binding domain of Xrp1 is required to mediate *caz* mutant phenotypes. (A)** Schematic representation of the Xrp1 transgene in the pUAST transgenesis vector, with five UAS GAL4-binding sites, and the pJFRC4 vector, with three UAS sites. **(B)** The Xrp1 AT-hook motif consists of nine amino acids including the invariant GRP triplet (top). To inactivate the DNA-binding capacity of the AT-hook motif, three amino acids essential for DNA binding (RGR) were mutagenized to alanine (bottom). **(C)** Panneuronal expression (*elav-GAL4*) of WT or AT-hook mutant (Mut) 3×UAS-Xrp1 transgenes (short isoform) does not induce developmental lethality. Adult offspring frequency relative to a driver-only control (100%) is shown. *n* > 376 per genotype. **(D and E)** Adult offspring frequency of *caz^2^, elav-GAL4/Y;; Df(3R)Xrp1^Plus^/+* (D) or *caz^KO^, elav-GAL4/Y;; Df(3R)Xrp1^Plus^/+* (E) males expressing 3×UAS-Xrp1 transgenes (WT or AT-hook mutant) or no transgene (control). *n* > 115 per genotype. ***, P < 0.0001; χ^2^ test.

As neuron-selective expression of Xrp1 from the standard pUAST transgenesis vector induces phenotypes by itself (Fig. 3, G–I; and Fig. S4, D–F), we used a modified pUAST vector with only three UAS sites, known to result in lower transgene expression levels (Fig. 6 A; Pfeiffer et al., 2010). The resulting 3×UAS-Xrp1 lines did not induce developmental lethality when selectively expressed in neurons (*elav-GAL4*; Fig. 6 C). Neuronal Xrp1 expression from 3×UAS transgenes was nevertheless able to revert the rescue of *caz* mutant pupal lethality by *Xrp1* heterozygosity (Fig. 6, D and E). We therefore inactivated the Xrp1 AT-hook domain in the 3×UAS constructs by mutagenizing the RGR triplet of the KRKRGRPAK motif to AAA, known to abolish AT-hook–mediated DNA binding (Fig. 6 B; Metcalf and Wassarman, 2006; Turlure et al., 2006; Baker et al., 2013). The subtle AT-hook mutation altered the binding pattern of Xrp1 on polytene chromosomes (Fig. S3 C) but did not reduce the stability of the Xrp1 protein and in fact increased Xrp1 protein level (Fig. S1, D–F). In spite of this, AT-hook–mutant UAS-Xrp1 transgenes were no longer able to revert the rescue of *caz* mutant pupal lethality by *Xrp1* heterozygosity (Fig. 6, D and E). These data demonstrate that the genetic interaction between *caz* and *Xrp1* is dependent on the functionality of the AT-hook DNA-binding motif in Xrp1.

### Gene expression dysregulation in *caz* mutants is rescued by *Xrp1* heterozygosity

FUS is known to be involved in transcriptional regulation and mRNA splicing (Schwartz et al., 2012, 2015; Tan et al., 2012; Yang et al., 2014), and knockdown or knockout of Fus in the mouse brain results in gene expression dysregulation (Ishigaki et al., 2012; Lagier-Tourenne et al., 2012; Scekic-Zahirovic et al., 2016). Furthermore, AT-hook proteins are often involved in gene expression regulation either as transcription factors or as chromatin architectural proteins (Reeves, 2010), and our results thus far indicate that Xrp1 is a nuclear chromatin-binding protein likely involved in gene expression regulation. We therefore hypothesized that loss of *caz* function may result in gene expression dysregulation, which could possibly be mitigated by heterozygosity for *Xrp1*.

**Figure 7.**
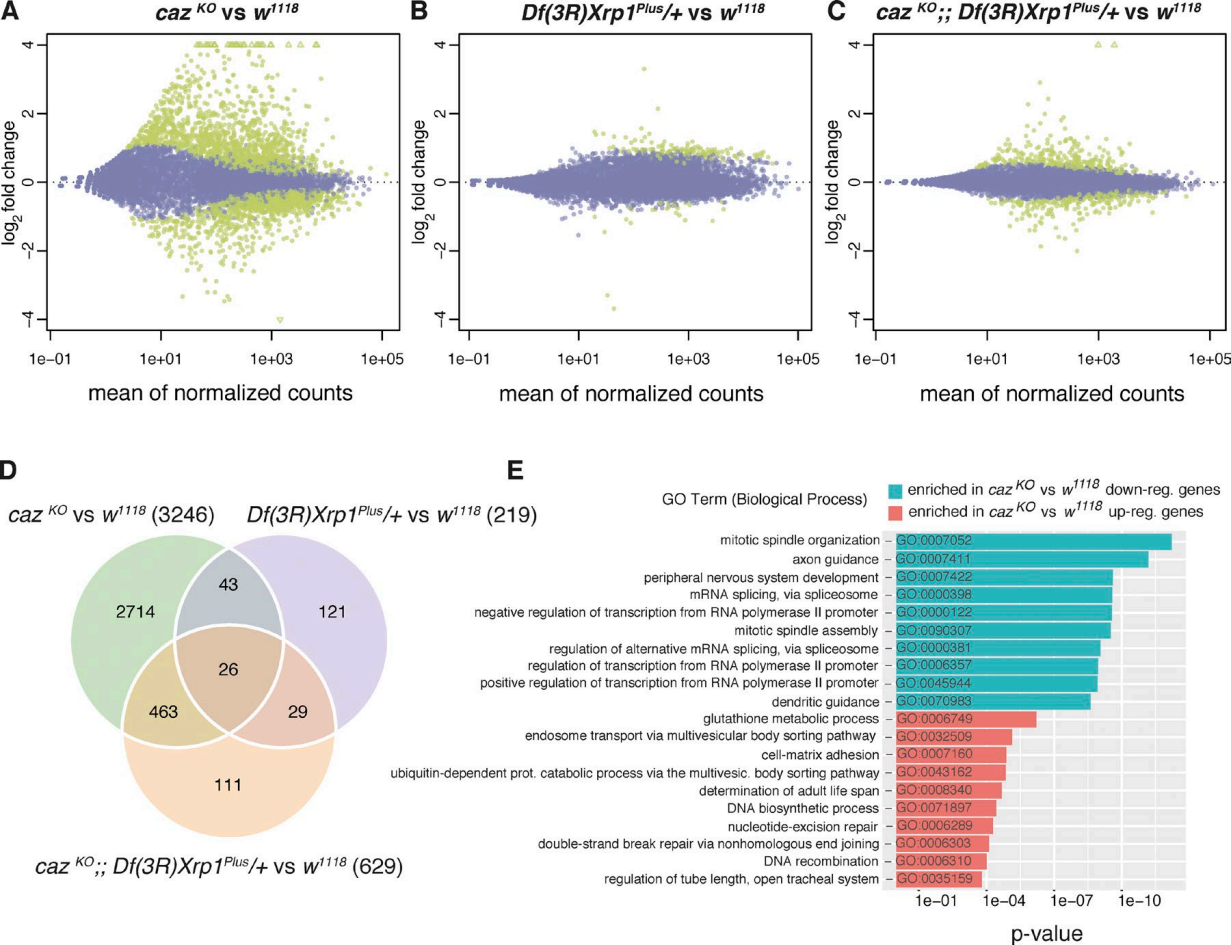
**Heterozygosity for *Xrp1* mitigates gene expression dysregulation in *caz* mutant CNS. (A–C)** MA plots displaying gene expression changes in *caz^KO^* versus *w^1118^* (genetic background control; A), *Df(3R)Xrp1^Plus^*/+ versus *w^1118^* (B), and *caz^KO^;; Df(3R)Xrp1^Plus^/+* versus *w^1118^* (C). X axes represent the mean of the normalized read counts per gene across all samples included in each comparison. Y axes represent the log_2_ fold change per gene resulting from each comparison. Green dots correspond with differentially expressed genes, with P < 0.05 adjusted for multiple testing. **(D)** Venn diagram representing the overlap between differentially expressed genes across the three comparisons. Numbers in parenthesis indicate the total number of differentially expressed genes in each comparison. **(E)** Top 10 enriched GO terms (Biological Process ontology) in the *caz^KO^* versus *w^1118^* comparison for the set of up-regulated (red) and down-regulated genes (blue).

To test this hypothesis, we used RNA sequencing (RNA-seq) to evaluate mRNA expression levels in third instar larval CNS of *caz^KO^* and *caz^KO^ Xrp1* heterozygous animals as well as *Xrp1* heterozygous and WT animals as controls. Principal component analysis and clustering of the samples discriminated the four genotypes from each other, with a certain degree of overlap between *Xrp1* heterozygous and WT samples (Fig. S5, A and B). Differential gene expression analysis between mRNA levels from WT and *caz^KO^* identified 1,641 up-regulated and 1,605 down-regulated genes in *caz* mutants (FRD-adjusted P value <0.05; Fig. 7, A and D; and Table S5), indicating substantial gene expression dysregulation. Gene expression changes for *caz* and *Xrp1* were consistent with the previously obtained qPCR data (Fig. S5 C). In contrast, comparison between WT and *Xrp1* heterozygotes identified only 184 up-regulated and 30 down-regulated genes (Fig. 7, B and D). Most interestingly, in *caz* mutant *Xrp1* heterozygous CNS, 315 up-regulated and 314 down-regulated genes were identified, with >90% of these displaying a less than twofold change (Fig. 7, C and D). Thus, heterozygosity for *Xrp1* significantly mitigated gene expression dysregulation in *caz* mutant CNS. Principal component analysis confirmed the dramatic gene expression dysregulation in *caz* mutant CNS, which was significantly rescued in *caz* mutant *Xrp1* heterozygous animals (Fig. S5 A).

Up- or down-regulation of a panel of 19 genes was validated by qPCR (Fig. S5 E), and gene ontology (GO) analysis showed that transcripts whose expression was altered in *caz* mutant animals were enriched for genes involved in processes such as axon and dendrite guidance, peripheral nervous system development, regulation of transcription and mRNA splicing, DNA repair, and mitotic spindle organization and assembly (Fig. 7 E). GO analysis for molecular function revealed that transcripts with altered expression in *caz* mutants were enriched for mRNAs encoding DNA- and chromatin-binding proteins as well as mRBPs, along with transcription factor activity, among others (Fig. S5 D). Overall, this is in line with the known functions of Caz and its mammalian FET protein orthologues (Schwartz et al., 2015) and strikingly similar to results from RNA-seq experiments in *Fus^−/−^* mice (Scekic-Zahirovic et al., 2016). In conclusion, our transcriptome analysis revealed that gene expression dysregulation in *caz* mutant CNS is substantially mitigated by *Xrp1* heterozygosity.

### Phenotypes induced by motor neuron–selective expression of ALS mutant FUS are substantially mitigated by Xrp1 knockdown

**Figure 8.**
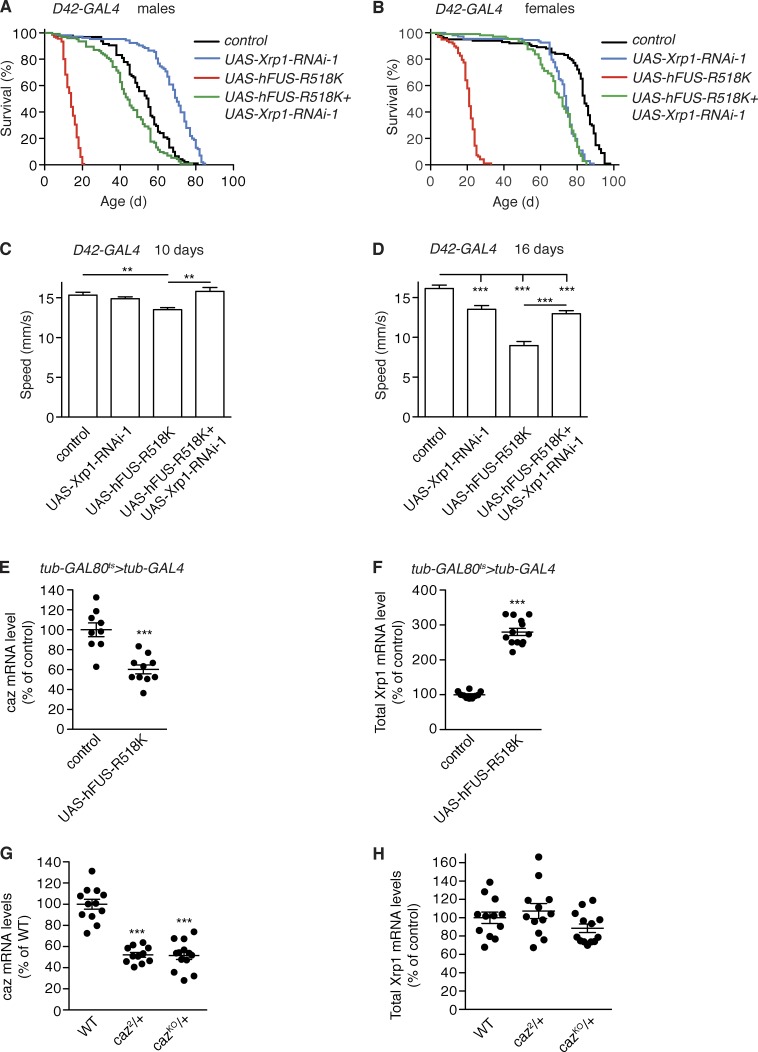
**Motor neuron–selective Xrp1 knockdown mitigates motor deficits and shortened life span induced by ALS mutant FUS expression. (A and B)** Life span of control (driver only) flies and flies with motor neuron–selective (*D42-GAL4*) expression of human FUS-R518K, Xrp1-RNAi, or both transgenes. Data for male (A) and female (B) flies are shown. *n* > 75 per genotype. **(C and D)** Average climbing speed of adult female flies with motor neuron–selective (*D42-GAL4*) expression of human FUS-R518K, Xrp1-RNAi, or both transgenes versus driver-only controls. Flies were tested at 10 (C) and 16 (D) d of age. *n* > 100 per genotype. **, P < 0.01; ***, P < 0.005; Mann-Whitney test. **(E and F)** Transcript levels of caz (E) and Xrp1 (F) in heads of adult female flies 3 d after induction of ubiquitous FUS-R518K expression (*tub-GAL4*) versus driver-only controls. *n* = 9–11. ***, P < 0.0005; two-tailed unpaired *t* test. **(G and H)** Caz (G) and Xrp1 (H) transcript levels in the CNS of third instar female larvae heterozygous for *caz^2^* or *caz^KO^* versus WT controls. *n* = 11–13. ***, P < 0.001; one-way ANOVA. All graphs display mean ± SEM.

To evaluate the potential relevance of our findings for human ALS, we used a *Drosophila* ALS-FUS model. Selective expression of R518K mutant human FUS in motor neurons (*D42-GAL4*) yielded adult flies that developed progressive motor deficits and displayed a substantially shortened life span (Fig. 8, A–D). We therefore evaluated the effect of Xrp1 knockdown on motor behavior and life span of these flies. Coexpression of Xrp1-RNAi more than tripled the median life span of both male and female *D42-GAL4*>UAS-FUS-R518K flies (Fig. 8, A and B). At 10 d of age, FUS-R518K flies displayed a mild climbing defect (reduction in speed by ∼12%), which was fully rescued by motor neuron–selective Xrp1 knockdown (Fig. 8 C). At 16 d of age, FUS-R518K flies displayed a severe climbing defect (reduction in speed by ∼45%), which was rescued by knockdown of Xrp1 to a level that was not significantly different from *D42-GAL4*>UAS-Xrp1-RNAi control flies (Fig. 8 D). Thus, reduction of Xrp1 levels in motor neurons substantially rescues the motor deficits and shortened life span of a *Drosophila* ALS-FUS model.

To gain insight into the mechanism underlying this major phenotypic rescue, we evaluated the effect of FUS-R518K overexpression on *caz* and *Xrp1* transcript levels. Ubiquitous overexpression of FUS-R518K in adult female flies moderately reduced caz transcript levels to ∼60% of control levels (Fig. 8 E). Strikingly, Xrp1 transcript levels were about threefold increased upon FUS-R518K expression (Fig. 8 F). This substantial increase in Xrp1 expression cannot be attributed to the moderate reduction in caz levels because Xrp1 transcript levels were not altered in *caz* heterozygous females in spite of a ∼50% reduction of caz transcript levels (Fig. 8, G and H). Thus, expression of ALS mutant FUS results in substantial up-regulation of Xrp1 expression independent of caz levels. The fact that Xrp1 knockdown substantially rescues phenotypes induced by ALS mutant human FUS indicates that these phenotypes are to a large extent mediated by increased Xrp1 expression.

## Discussion

**Figure 9.**
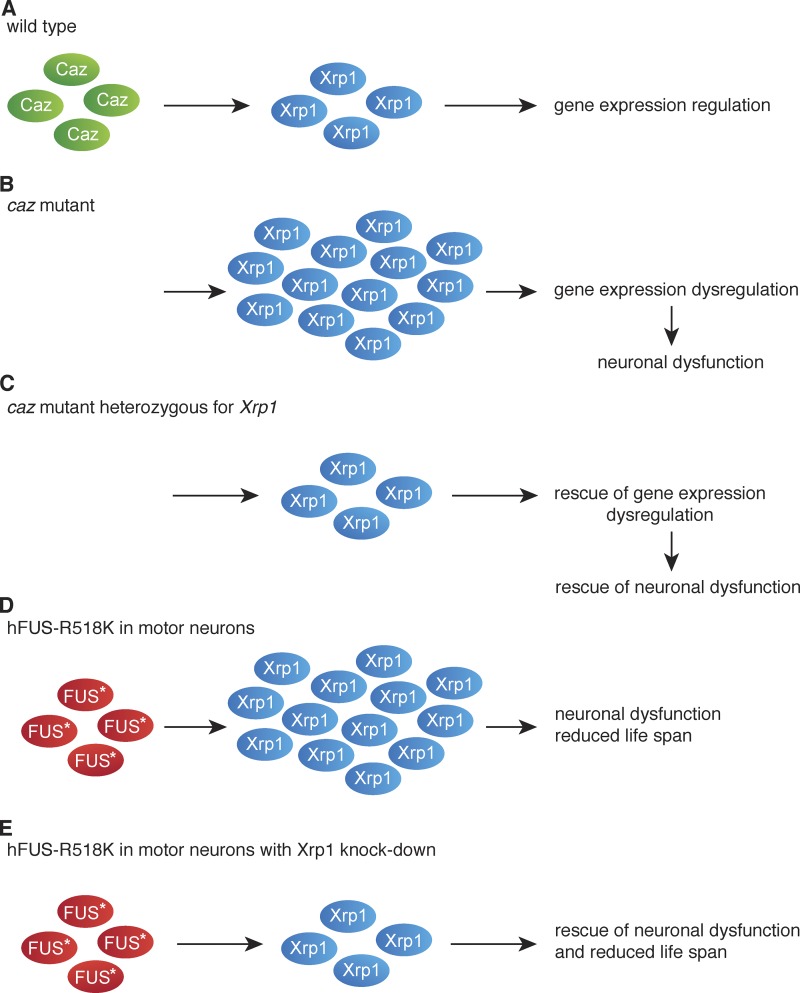
**Xrp1 is a key mediator of *caz* mutant phenotypes. (A)** In WT animals, Caz controls Xrp1 levels, resulting in normal gene expression regulation. **(B)** Loss of *caz* function results in substantial up-regulation of *Xrp1* expression, leading to gene expression dysregulation and neuronal dysfunction. **(C)** In *caz* mutant *Xrp1* heterozygous animals, *Xrp1* levels are normalized, resulting in rescue of gene expression dysregulation and neuronal dysfunction. **(D)** Expression of ALS mutant human FUS results in substantial up-regulation of *Xrp1* expression, and motor neuron–selective expression induces neuronal dysfunction and reduced life span. **(E)** Simultaneous knockdown of Xrp1 in motor neurons expressing FUS-R518K rescues neuronal dysfunction and reduced life span.

In this study, we identified *Xrp1* as a genetic modifier of *caz* mutant phenotypes. *Caz* is the single *Drosophila* orthologue of the three human FET family proteins FUS, EWSR1, and TAF15 (Schwartz et al., 2015). *Xrp1* expression was up-regulated by three- to fourfold in *caz* mutant animals, and heterozygosity for *Xrp1* fully rescued the *caz* mutant eclosion defect and partially but substantially rescued adult motor performance and life span. Exhaustive genetic screening of the second and third chromosome, which together constitute ∼80% of the fly genome, identified *Xrp1* as the only gene for which reduction of gene dosage by 50% could rescue caz mutant pupal lethality, indicating that *Xrp1* is a key modifier of *caz* mutant phenotypes. Interestingly, in spite of the previously reported ubiquitous expression of *Xrp1* (Tsurui-Nishimura et al., 2013) and the fact that Xrp1 expression was not only increased in the CNS but also in the body wall and presumably other tissues of *caz* mutants, neuron-selective knockdown of *Xrp1* was sufficient to rescue *caz* mutant phenotypes, and selective neuronal overexpression of Xrp1 in otherwise WT animals phenocopied *caz* mutant phenotypes. This is consistent with the previously reported key function of Caz in neurons (Wang et al., 2011; Frickenhaus et al., 2015). Together, our data indicate that *caz* mutant phenotypes are largely mediated by increased *Xrp1* expression, with particularly deleterious effects in neurons (Fig. 9).

*Xrp1* has previously been implicated in protection against genotoxic stress and DNA damage repair (Brodsky et al., 2004; Akdemir et al., 2007; Francis et al., 2016). Consistently, a number of Xrp1-interacting proteins are involved in DNA repair (Fig. 5 C), and GO analysis of our RNA-seq data revealed enrichment for genes involved in DNA repair among genes up-regulated in *caz* mutant CNS (Fig. 7 E). This study revealed a novel role for Xrp1 in gene expression regulation as a substantial number of Xrp1-interacting proteins are involved in regulation of transcription, chromatin organization, and RNA metabolism (Fig. 5 C). Interestingly, neuronal overexpression of the long Xrp1 isoform induced significantly stronger phenotypes as compared with the short isoform (Fig. 3, G–I; and Fig. S4, D–F), despite insertion of the transgenes in the same genomic site and similar expression levels (Fig. 3 B). This is likely attributable to the additional N-terminal 262 aa of the long isoform, allowing the binding of substantially more interacting proteins (Fig. 5 B and Table S2), which may result in more pronounced gene expression dysregulation and stronger phenotypes.

Consistent with a key role of Xrp1 in gene expression regulation, significant gene expression dysregulation was found in *caz* mutant CNS, which was substantially mitigated by normalizing Xrp1 levels in *caz* mutants (Fig. 7). Importantly, of the 3,246 differentially expressed genes in *caz* mutants, only 489 are still significantly up- or down-regulated *caz* mutant *Xrp1* heterozygotes (Fig. 7 D). The 2,757 genes that are significantly changed in *caz* mutants but not in *caz* mutant *Xrp1* heterozygotes are likely direct or indirect targets of Xrp1, and up- or down-regulation of these genes in *caz* mutants may contribute to neuronal dysfunction. The novel function of Xrp1 in gene expression regulation is likely dependent on the capacity of Xrp1 to bind DNA, presumably mediated by two predicted DNA-binding domains in its C terminus: an AT-hook motif and a bZIP motif. Whereas the functionality of the predicted bZIP motif remains to be investigated, the AT-hook motif of Xrp1 conforms with the consensus sequence, consisting of nine amino acids centered on the invariant tripeptide glycine-arginine-proline (Reeves, 2010). The DNA-binding capacity of the Xrp1 AT-hook motif is likely required to mediate gene expression regulation and dysregulation, as the introduction of a subtle mutation in this motif demonstrated that its functionality is essential to mediate *caz* mutant phenotypes. Based on our findings, we propose a working model in which *caz* mutant phenotypes are mediated by increased *Xrp1* expression, leading to gene expression dysregulation and neuronal dysfunction (Fig. 9).

Extensive bioinformatic searches did not reveal a clear one-to-one *Xrp1* orthologue in mammals. However, we believe that it is highly likely that *Xrp1* has functional homologues in mammals. Candidate functional homologues include 27 human genes encoding proteins predicted to contain at least one AT-hook motif (Table S6), including the Rett syndrome gene *MECP2* (Amir et al., 1999). Interestingly, DNA binding mediated by the MeCp2 AT-hook domains has been implicated in the pathogenesis of Rett syndrome (Baker et al., 2013), and FUS was reported to bind the MECP2 promotor and positively regulate MECP2 transcription (Tan et al., 2012). In addition, FUS also binds MECP2 mRNA (Lagier-Tourenne et al., 2012), indicating that MECP2 is both a transcriptional and mRNA target of FUS. Furthermore, brains from *Fus^−/−^* mice or transgenic mice overexpressing ALS mutant FUS display up-regulation of *Cbx2*, *Dot1l*, *Elf3*, *Prr12*, and *KMT2B* (Scekic-Zahirovic et al., 2016; Shiihashi et al., 2016), and 17 of the 27 AT-hook genes are reported FUS RNA targets (Table S6). En route toward identification of human functional homologues of Xrp1, it will be particularly important to gain detailed molecular insight into how Xrp1 regulates gene expression. A first step could be the identification of the genomic binding sites of Xrp1 and its putative target genes. Furthermore, since Xrp1 does not have other predicted functional domains apart from the AT-hook and bZIP domains, it is tempting to speculate that Xrp1 regulates gene expression by recruiting other proteins that contain functional domains such as transactivation or histone-modifying domains to specific genomic sites. Several of the Xrp1-interacting proteins identified in this study contain such functional domains and have human orthologues (e.g., TAF9, ZMYM2, NFX1, HCFC1/2, VRK1, RSF1, and BPTF).

Interestingly, gene expression dysregulation was previously implicated in ALS and FTD pathogenesis. For instance, a significant enrichment in de novo mutations in the chromatin regulatory pathway in sporadic ALS patients was reported (Chesi et al., 2013). Furthermore, involvement of the three FET proteins in regulation of transcription and mRNA splicing is well established (Schwartz et al., 2015), and in fact, several other RBPs that have been implicated in ALS and FTD pathogenesis are also known to play important roles in gene expression regulation (Ling et al., 2013). These include TDP-43, involved in regulation of transcription and mRNA splicing (Buratti and Baralle, 2010), which mislocalizes to cytoplasmic inclusions with nuclear clearance in >95% of ALS and ∼45% of FTD patients (Ling et al., 2013). Furthermore, the ALS-causing expanded hexanucleotide repeat in *C9orf72* may sequester RBPs, thus inducing gene expression dysregulation (Lee et al., 2013; Haeusler et al., 2014). The potential relevance of our findings for ALS-FUS pathogenesis is further indicated by the fact that knockdown of *Xrp1* substantially rescues the motor deficits and shortened life span of flies that selectively express ALS mutant human FUS in motor neurons (Figs. 8 and 9). This may be explained by a substantial increase in Xrp1 expression induced by mutant FUS, which is not attributable to the moderate (∼40%) down-regulation of *caz* expression.

Finally, our findings may also be relevant for FTLD-FUS pathogenesis as this disease is characterized by pathological inclusions containing not only FUS but also TAF15 and EWS, with reduced levels or complete loss of nuclear FET proteins in inclusion-bearing neurons and glial cells (Neumann et al., 2011; Davidson et al., 2013). Thus, loss of FET protein function and consequent gene expression dysregulation may contribute to FTLD-FUS pathogenesis. Consistently, FUS knockout or knockdown in the hippocampus of mice induces behavioral aberrations related to FTD symptoms (Kino et al., 2015; Udagawa et al., 2015). In conclusion, our findings provide important novel insights into the molecular mechanisms by which loss of Caz, the *Drosophila* orthologue of human FET proteins, induces motor deficits and reduced life span, and they suggest that gene expression dysregulation may be involved in the pathogenesis of human FUSopathies.

## Materials and methods

### *Drosophila* genetics

Flies were housed in a temperature-controlled incubator with 12:12 h on/off light cycle at 25°C, and for some experiments, at 23°C (5×UAS-Xrp1 overexpression), in vials containing standard cornmeal medium. X chromosome–inserted *elav-GAL4* (458; Bloomington Drosophila Stock Center [BDSC]) was used for panneuronal expression of UAS transgenes, *OK371-GAL4* and *D42-GAL4* were used for targeted expression in motor neurons, and *tub-GAL4* was used for ubiquitous expression of UAS transgenes.

For the dominant suppressor screen, deficiencies covering the second and the third chromosome from the Bloomington Deficiency Kit were used as this kit provides maximal coverage (euchromatic coverage ≥ 97.5%) with a minimal number of deletions (Cook et al., 2012). Df/Balancer males were crossed to *caz^2^/FM7* females to screen for the emergence of *caz^2^*/Y; Df/+ males in the offspring, which would indicate suppression of pupal lethality of *caz^2^* males by hemizygosity for the deficiency. To narrow down the genomic region that is uncovered by *Df(3R)ED2*, the deficiency that mediated rescue of *caz* mutant pupal lethality, molecularly mapped smaller deficiency lines in this region were ordered from the BDSC. PCR genotyping of *caz* mutant males was used to exclude X chromosome nondisjunction in all experiments.

The UAS-Xrp1-RNAi lines used in this study were P[TRiP.HMS00053]attP2 (34521; BDSC; UAS-Xrp1-RNAi-1) and P[GD9476]v33010 obtained from the Vienna Drosophila Resource Center (UAS-Xrp1-RNAi-2). The UAS-FUS-R518K transgenic line was generated by and obtained from Lanson et al. (2011).

### Generation of *Xrp1* mutant lines

For generation of Xrp1 deletion lines, *P[XP]d11439*, *P[XP]Xrp1^d04790^*, *PBac[WH]f07598*, and *PBac[WH]f05721* were obtained from the Drosophila Genetic Resource Center at the Kyoto Institute of Technology and used to isolate *Xrp1* chromosomal deletions (Fig. 1, D and E) following the basic schemes outlined by Parks et al. (2004). The chromosomal deletions generated were verified by PCR with primers flanking the transposable element insertions (Table S7), followed by sequencing of the PCR fragments.

For generation of a clean *Xrp1*-null allele, in vivo homologous recombination was used to target the *Xrp1* gene. Following a previously published strategy (Vilain et al., 2014), the presence of a Mi{MIC} transposon in the *Xrp1* gene (Mi{MIC}Xrp1^MI07118^) was exploited for site-specific insertion of a targeting construct (Figs. 1 and S1 C). For the construction of a targeting vector, a fosmid (FlyFos clone number FF017187) containing the extended Xrp1 genomic region was used to PCR amplify left and right homology arms using the primers Xrp1_LHA_FW, Xrp1_LHA_REV, Xrp1_RHA_FW, and Xrp1_RHA_REV (Table S7). To minimize the chance of introducing mutations during PCR amplification, Phusion high-fidelity DNA polymerase (New England Biolabs) was used with only 20 cycles of PCR amplification. The obtained PCR products were subcloned in a Zero Blunt TOPO PCR cloning vector (Invitrogen) and sequence verified. The presence of a HindIII site in primer Xrp1_LHA_FW and an EcoRI site in primer Xrp1_LHA_REV was subsequently used to clone the left homology arm into pABC (Choi et al., 2009). Next, the presence of an EcoRI site in Xrp1_RHA_FW and a KpnI site in Xrp1_RHA_REV was used to clone the right homology arm into the pABC vector that already contained the left homology arm.

The obtained targeting vector was sequence verified and injected into Mi{MIC}Xrp1^MI07118^ embryos for site-specific integration of the targeting construct into the Mi{MIC} transposable element in the Xrp1 gene. Transgenic lines in which the transgenic construct was integrated into Mi{MIC}Xrp1^MI07118^ in the correct orientation were identified. These lines were subsequently crossed to a transgenic line that expresses I-SceI under the control of a heat-inducible promoter. Given the presence of an I-SceI restriction site in primer Xrp1_LHA_FW, this will induce a double-strand break adjacent to the left homology arm of the targeting construct, allowing for precise removal of the Xrp1 and Mi{MIC} sequences left of the targeting construct through homologous recombination (Fig. S1 C). Lines with successful homologous recombination were identified by PCR and sequencing of the obtained PCR fragments. Next, these lines were crossed to a transgenic line that expresses I-CreI under the control of a heat-inducible promotor. Given the presence of an I-CreI restriction site in primer Xrp1_RHA_REV, this will induce a double-strand break adjacent to the right homology arm of the targeting construct, allowing for precise removal of the Xrp1 and Mi{MIC} sequences right of the targeting construct through homologous recombination. Lines with successful homologous recombination were identified by PCR and sequencing of the obtained PCR fragments.

### Generation of UAS-Xrp1 transgenic lines

For generation of UAS-Xrp1^Long^ transgenic lines, RNA was extracted from WT flies and converted into cDNA, which was used as a template for PCR (primer sequences in Table S7) to amplify the transcript coding for the long Xrp1 isoform. Gold clone FI10013 containing the Xrp1^Short^ cDNA was obtained from Kyoto Stock Center. Xrp1^Long^ and Xrp1^Short^ cDNAs were subsequently cloned into pUAST-attB using either NotI or EagI as well as XhoI restriction sites (Table S7). Site-directed PCR mutagenesis was used to generate AT-hook mutant versions of the long and short Xrp1 isoforms (mutagenesis primers are included in Table S7). WT and AT-hook mutant Xrp1 cDNAs were subsequently amplified by PCR using Phusion high-fidelity DNA polymerase (New England Biolabs) and primers containing XhoI and XbaI restriction sites (Table S7). The obtained PCR products were subcloned in a Zero Blunt TOPO PCR cloning vector (Invitrogen), and XhoI and XbaI were used to transfer the Xrp1 cDNAs to the pJFRC4 vector, which contains three UAS sites (Pfeiffer et al., 2010). UAS constructs were embryo injected following standard procedures. For each of the constructs, VK31 (on III) and VK37 (on II) genomic landing sites were used to avoid any influence of neighboring genomic sequences on transgene expression. As neuronal expression of 5×UAS-Xrp1 transgenes (*elav-GAL4*) in many cases resulted in developmental lethality with no adult escapers when raised at 25°C, experiments in which *elav-GAL4* was used to drive expression of 5×UAS-Xrp1 transgenes were performed at 23°C.

### Motor performance assay

For assaying mobility, flies were collected within 24 h after eclosion and divided into groups of 10 individuals. Motor performance of 3-, 10-, or 16-d-old flies was evaluated as described earlier (Frickenhaus et al., 2015; Niehues et al., 2015), and average climbing speed (mm/s) was determined and compared between genotypes. As female *D42-GAL4*>FUS-R518K flies lived longer than males, we studied the effect of Xrp1 knockdown on age-dependent motor deficits in female flies.

Larval locomotion was analyzed using the frustrated total internal reflection–based imaging method FIM (Risse et al., 2013, 2014, 2017). Batches of 15 third instar larvae were allowed to freely move for 3 min on a recording platform at RT. Tracking data were obtained using FIMTrack (http://fim.uni-muenster.de), and output files were analyzed with MatLab (MathWorks). In Fig. S3 B, larvae were sorted in a Petri dish with water for ∼5 min, and only male larvae were recorded.

### Adult offspring frequency and life span analysis

For determination of adult offspring frequencies, appropriate crosses were set up, and the number of adult flies eclosing was counted for each genotype. For life span analysis, newly eclosed flies were collected and housed at a density of 10 flies per vial. At least 75–100 flies were tested for each genotype. The number of dead flies was counted every day, and the flies were transferred to fresh food vials every 2–3 d.

### Real-time qPCR

Total RNA was extracted from 15–20 third instar larval brains or from four adult male flies per biological replicate using NucleoSpin RNA (Macherey-Nagel) according to the manufacturer’s instructions. Reverse transcription was performed on 1 µg RNA treated with gDNA Wipeout Buffer using the Quantitect Reverse Transcription kit (QIAGEN). Resulting cDNA samples were used as templates for real-time PCR assays performed on an ABI 7300 system (Applied Biosystems) with iTaq Universal SYBR Green supermix (Bio-Rad Laboratories). Primers used for quantitation of caz and Xrp1 transcript levels are listed in Table S7. Measurements were normalized to *EifTuM* and *rp49* controls. Data were analyzed using the ΔΔCt calculation method. Experiments included no–reverse transcriptase controls for each template and no-template controls for each pair of primers.

### Western blotting

For Western blots, protein extracts were made by homogenizing third instar larval CNS in extraction buffer (50 mM Tris/HCl, pH 7.4, 150 mM KCl, 0.25 M sucrose, 5 mM MgCl_2_, and 0.5% Triton X-100). Lysates of *Drosophila* S2 cells transfected with plasmids encoding *actin5C-GAL4* alone or cotransfected with plasmids encoding N-terminal HA-tagged Xrp1^Short^ or Xrp1^Long^ were prepared in cell lysis buffer (20 mM Tris, 200 mM NaCl, 1 mM EDTA, and 0.5% NP-40 containing 1 U complete mini protease inhibitor cocktail [Roche]). Samples separated on 10% SDS-PAGE were electrotransferred onto polyvinylidene difluoride (EMD Millipore) for 45 min at 15 V. Blotted membranes were incubated overnight at 4°C with primary antibodies against Caz (mouse monoclonal 3F4; 1:30; Immanuel et al., 1995), HA epitope tag (mouse monoclonal HA.11; 1:1,000; Covance), and β-tubulin (mouse monoclonal E7; 1:700; Developmental Studies Hybridoma Bank). Immunoreactive proteins were visualized after incubation with anti-mouse IgG coupled to horseradish peroxidase (W402B; 1:2,500; Promega) for 1 h at RT. Blots were developed with enhanced chemiluminescence (GE Healthcare), and x-ray film images of chemiluminescence were developed and scanned. Densitometric quantification of images was performed with ImageJ/FIJI (National Institutes of Health).

### Coimmunoprecipitation

For detection of potential homodimers of Xrp1^Short^ and Xrp1^Long^, *Drosophila* S2 cells were either transfected with a plasmid encoding *actin5C-GAL4* alone or cotransfected with plasmids encoding N-terminal HA- or Flag-tagged Xrp1^Short^ or Xrp1^Long^ constructs, all under UAS control. 48 h after transfection, protein lysates of cells expressing HA- or Flag-tagged Xrp1 were either directly used or combined in a 1:1 ratio. 5% of protein extracts were used for Western blotting, while the remaining 95% were added to anti-HA agarose beads for 24 h at 4°C. Immunoprecipitation of HA-tagged proteins was performed using an Anti-HA Immunoprecipitation Kit (Sigma-Aldrich), according to the manufacturer’s protocol.

To evaluate whether Caz and Xrp1 physically associate with each other, S2 cells expressing the *actin5C-GAL4* plasmid alone (control) or in conjunction with the N-terminal HA-tagged Xrp1^Short^ or Xrp1^Long^ constructs were seeded at a density of 10^6^ cells/ml in 1 ml Shields and Sang medium (Sigma-Aldrich) in 12-well plates 1 d before transfection. Transfection was performed using Fugene HD Transfection Reagent (Promega) according to manufacturer’s instructions. Cell lysates were prepared, cellular debris was pelleted by centrifugation at 12,000 *g* for 15 min and washed, and the protein-containing supernatant was incubated overnight at 4°C with 100 µl of either anti-Flag (10 µg; clone M2; F1804; Sigma-Aldrich) or anti-Caz (10 µg; 3F4; Immanuel et al., 1995) conjugated SureBeads Protein G Magnetic Beads (Bio-Rad Laboratories) according to the manufacturer’s instructions. Bound proteins were eluted by heating to 70°C for 10 min with 40 µl of 1× Laemmli buffer (Bio-Rad Laboratories).

Inputs, precipitates, and binding proteins were analyzed by SDS-PAGE and immunoblotting. The immunoblot analyses were performed using the following primary antibodies: anti-Flag (clone M2; F1804; 1:1,500; Sigma-Aldrich) and anti-HA (Mono HA.11; 1:1,000; Covance).

### Liquid chromatography (LC)–tandem MS analysis

S2 cells were seeded at a density of 10^6^ cells/ml in 3 ml Shields and Sang medium (Sigma-Aldrich) in six-well plates 1 d before transfection. Flag-tagged Xrp1^Short^ or Xrp1^Long^ plasmids were transfected, along with the *actin5C-GAL4* construct. Three replicates were processed for each condition. After incubating the cells for 2 d with the transfection mixes, cells were collected and lysed in cell lysis buffer (described above). Cellular debris was cleared by centrifugation at 12,000 *g* for 15 min. To pull down Xrp1-interacting proteins, the protein-containing supernatant was applied to 100 µl anti-Flag (clone M2; F1804; Sigma Aldrich)–conjugated SureBeads Protein G Magnetic Beads (Bio-Rad Laboratories) according to the manufacturer’s instructions. Cell lysates were incubated with the antibody beads overnight at 4°C. Washed beads were resuspended in 4% SDS and 50 mM Tris, pH 7.5, and bound proteins were eluted by heating to 95°C for 10 min and then precipitated with a fourfold excess (vol/vol) of ice-cold acetone overnight to remove detergent and salts. Precipitated protein pellets were washed twice with 90% acetone, air dried, and then resuspended in 8 M urea and 50 mM Tris-HCl, pH 8.5, before in-solution digestion, first with endopeptidase LysC (1 µg/immunoprecipitation) for 3 h at 37°C, and then with trypsin overnight at 37°C (1.5 µg/immunoprecipitation). After acidification of the digest by addition of 1% formic acid (final concentration), peptides were desalted using Empore-C18 StageTips (Rappsilber et al., 2003) and stored at 4°C until further use. Prior to LC–tandem MS, peptides were eluted using 2 × 20 µl of 80% acetonitrile and 0.1% formic acid, and then they were dried in an Eppendorf concentrator to a volume of ∼2 µl and resuspended in 10 µl buffer A (0.1% acetic acid). 6 µl of this peptide solution was then analyzed by nanoscale reverse-phase chromatography using an EASY nLC 1200 (Thermo Fisher Scientific) as a high-performance LC pump and a Picofrit column (25 cm × 75 µm ID; New Objective) filled with C18 reverse-phase material (Reprosil pur C18-AQ; 1.9 µm; Dr. Maisch GmbH) that was online coupled via a Nanospray Flex electrospray ionization source (Thermo Fisher Scientific) to a QExactive HF mass spectrometer (Thermo Fisher Scientific). Peptides were separated at a flow rate of 300 nl/min using a gradient running from 3–35% B (80% acetonitrile and 0.1% formic acid) in 90 min, which was ramped up to 100% B in 5 min, where it was maintained for additional 10 min before reequilibration at starting conditions. Column temperature was maintained at 45°C with the help of a column oven (PRSO-V1; Sonation). The mass spectrometer was operated in data-dependent mode, acquiring full-scan spectra in profile mode at a resolution of 60,000 and an automatic gain control target value of 3 × 10^−6^ (scan range 300–1,650 m/z). Spray voltage was set to 2.1 kV. The 17 most intense ions were chosen for higher energy collisional dissociation with a resolution of 15,000 at m/z 200 and a target value of 10^−5^. The isolation window was set to 1.6 m/z, and the normalized collision energy to a value of 28. Dynamic exclusion was allowed and set to 20 s. Uncharged as well as singly charged compounds were excluded from the analysis as well as peptides with a charge state >6. Data were recorded with Xcalibur software (Thermo Fisher Scientific).

### MS data analysis

Raw MS files were processed using the MaxQuant computational platform (version 1.5.3.8; Cox and Mann, 2008). The Andromeda search engine integrated into MaxQuant was used for the identification of peptides and proteins by querying a concatenated forward and reverse UniProt *Drosophila* database (UP000000803_7227.fasta; release 2015-12), including common laboratory contaminants. The search for precursor and fragment ions was performed allowing an initial mass deviation of 20 ppm and 0.5 D, respectively. Trypsin with full enzyme specificity was selected, and only peptides with a minimum length of seven amino acids were allowed. A maximum of two missed cleavages was allowed. Carbamidomethylation (Cys) was set as fixed modification, while oxidation (Met) and N-acetylation were defined as variable modifications. For protein and peptide identification, a minimum false discovery rate of 1% was required. Label-free quantification (LFQ) was based on the measurements of three independent biological replicates for each strain analyzed by the MaxQuant LFQ algorithm with the “match between runs” option turned on (Cox et al., 2014). Further data processing was performed using the bioinformatics module Perseus (version 1.5.6.0; Cox et al., 2011). Following initial filtering and grouping (actin; caz; XRP1-L; XRP1-s), LFQ values were log_2_ transformed, and only proteins were included in the analysis that were identified with at least three valid values in at least one of the four groups. Still-missing values (NaN) were replaced by imputation, simulating signals of low abundant proteins within the distribution of measured values. A width of 0.3 SD and a downshift of 1.8 SD were used for this purpose. To identify proteins that displayed significant differences between the groups, ANOVA testing was performed (P = 0.05). Fold enrichment was calculated based on LFQ intensity values. The MS proteomics data have been deposited to the public PRIDE repository (Vizcaíno et al., 2013) via the ProteomeXchange platform (http://proteomecentral.proteomexchange.org) with the dataset identifier PXD008417.

### Immunocytochemistry and histochemistry

*Drosophila* S2R^+^ cells were seeded in a density of 4 × 10^5^ cells/ml on Concanavalin A (Sigma-Aldrich)–treated circular microscope cover glasses (12 mm; VWR) in a 24-well cell culture dish. After 24 h at 25°C, cells were transfected with a mix containing the Fugene HD transfection reagent (Promega) and *actin5C-GAL4* and UAS-HA::Xrp1^Short^ plasmids. 48 h later, cells were fixed in 4% PFA for 15 min followed by two 5-min washes in DPBS (1×; Gibco) at RT. After permeabilization with DPBS (1×) and 0.5% Triton X-100, cells were washed twice with DPBS. Cells were blocked for 1 h in 2% BSA and 10% goat serum in DPBS, followed by overnight incubation at 4°C with primary antibodies against Caz (mouse monoclonal clone 3F4; 1:30; Immanuel et al., 1995), lamin (mouse monoclonal ADL67.10; 1:100; Developmental Studies Hybridoma Bank), and HA (rabbit polyclonal; 1:50; Santa Cruz Biotechnology) diluted in 2% BSA and 10% goat serum in DPBS. After two washes in DPBS, secondary goat anti-mouse and anti-rabbit antibodies (Alexa Fluor 488 and 568; 1:500) were applied for 2 h at RT, followed by three washes in DPBS and mounting on microscopy slide with Aqua-Poly/Mount (Polysciences, Inc.).

For subcellular localization of Xrp1 in motor neurons, brains/CNS from wandering third instar larvae expressing *OK371-GAL4*>UAS-mCD8::GFP alone (control) or in conjunction with UAS-Xrp1^Short^ were dissected in PBS and fixed in 4% PFA for 30 min. Tissues were washed 3 × 10 min in PBS/0.2% Triton X-100 and blocked for 1 h at RT in 10% goat serum in PBS. Tissues were incubated with primary antibodies against Caz (mouse monoclonal clone 3F4; 1:30; Immanuel et al., 1995), lamin (mouse monoclonal ADL67.10; 1:100; Developmental Studies Hybridoma Bank), or HA (rabbit polyclonal; 1:50; Santa Cruz Biotechnology) overnight at 4°C with gentle agitation. Appropriate secondary antibodies conjugated either with Alexa Fluor 405, Alexa Fluor 488, or Alexa Fluor 568 (Molecular Probes) were used to detect the given primary antibody. All images were acquired using ZEN 2010 software on a Zeiss LSM700 laser scanning confocal microscope using an EC Plan neofluar 1.3 NA 40× oil-immersion objective.

### Squash preparation of polytene chromosomes from larval salivary glands

For preparing polytene chromosome squashes, salivary glands of WT third instar larvae were dissected in PBS (1×) and transferred to 1% Triton X-100 for 30 s. Fixation was in 4% PFA (1 min) and in 45% acetic acid/4% PFA (2 min). The glands were then incubated in 45% acetic acid (1 min) and subsequently squashed in the same solution under a coverslip to get polytene spreads. After freezing the slides in liquid nitrogen, coverslips were flipped off with a sharp blade, and slides were stored in 90% ethanol.

For immunostaining, squash preparations were rehydrated twice for 5 min in PBS (1×). Immunostaining was performed following the Dangli and Bautz (1983) procedure using rabbit polyclonal anti-Xrp1 (1:50; Francis et al., 2016) followed by an Alexa Fluor 568–conjugated secondary antibody (1:500; Molecular Probes). The preparation was counterstained with DAPI and mounted in Vectashield antifade mounting medium (Vector Laboratories) for confocal microscopy. All images were acquired using ZEN 2010 software on a Zeiss LSM700 laser scanning confocal microscope using an EC Plan neofluar 1.3 NA 40× oil immersion objective.

### RNA-seq and data analysis

For RNA-seq, total RNA was extracted from 15–20 brains dissected from wandering third instar male larvae using NucleoSpin RNA (Macherey-Nagel) according to the manufacturer’s instructions. After performing quality control checks, the RNA was sent to the Max Planck Genome Center, where cDNA libraries were prepared using the NEBNext Ultra Directional RNA Library Prep Kit for Illumina (New England Biolabs) using standard procedures. The libraries were sequenced on an Illumina HiSeq 2500 instrument as 100-bp paired-end reads each according to the manufacturer’s standard protocols. Three biological replicates per genotype were sequenced, with an average of 8.6 million nonredundant read pairs uniquely mapped to the *Drosophila* genome.

Preprocessing filtering of the reads before alignment, e.g., quality- or adapter-trimming, was not necessary. The *Drosophila* reference genome was downloaded from Flybase. Revision 6.04 of the genome assembly and gene annotation was used for all analyses. We aligned reads to the reference transcriptome using the TopHat pipeline (version 2.0.14; Kim et al., 2013a) with Bowtie2 (version 2.2.5; Langmead and Salzberg, 2012) and the flags b2-very-sensitive and library-type = fr-firststrand. The mapped reads were assigned to genes using the HTseq-count script from the HTseq package (Anders et al., 2015). We used the intersection-nonempty mode to exclude ambiguous gene assignments. Aligned pairs with a mapping quality <10 were excluded, and rRNA genes were removed from the gene list for further analysis.

Differential gene expression analysis was performed using DeSeq2 (version 1.11; Love et al., 2014). All comparisons were performed in a pairwise manner, comparing samples of each genotype separately against the WT. We chose to disable filtering genes based on Cook’s distance for the whole analysis because we observed that the high biological variability of *Xrp1* heterozygous animals lead to the exclusion of a large number of genes. Genes were called differentially expressed if the log_2_ fold change differed significantly from 0 with a false discovery rate–adjusted P value of <0.05. Expression levels for each annotated protein-coding gene were determined by the number of mapped reads per kilobase of exon per million mapped reads (RPKM).

Principal component analysis and hierarchical clustering of the global expression profile was performed on a variance-stabilized transformation of the read counts per gene using methods provided by the DeSeq2 R-package. For clustering, the distances between samples were calculated using the Manhattan distance metric. Based on the distance matrix, hierarchical clustering by complete linkage was performed using standard R functions.

GO-term enrichment analysis was done using the topGO package for R (version 2.22; Alexa et al., 2006). We extracted the sets of up- and down-regulated genes for each comparison from the DeSeq2 analysis and used the “weight01” algorithm in topGO in combination with Fisher’s exact test to check for enrichment of specific GO terms in these gene sets.

### Statistical analysis

χ^2^ statistics were used to analyze offspring frequency data. For life span analysis, the log-rank test was used to test for statistical significance. Motor performance was analyzed using the Mann-Whitney *U* test to compare climbing speed of individual flies per genotype and per run. As all flies were tested in three independent runs, three P values were generated per genotype. These P values were combined using the Fisher’s combined probability test. To analyze larval locomotion data, Mann-Whitney rank-sum tests were performed with MatLab. One-way ANOVA with Bonferroni correction was used to analyze Caz and Xrp1 mRNA and protein levels as data displayed normal distribution and equal variance. All images were assembled in figure panels using the Adobe Illustrator CS5 software.

### Online supplemental material

Fig. S1 shows generation and characterization of *Xrp1* mutant and transgenic lines. Fig. S2 shows that heterozygosity for *Xrp1* does not rescue the adult eclosion defect of *TBPH* mutant flies. Fig. S3 shows larval locomotion phenotypes and binding of WT or AT-hook mutant Xrp1 to polytene chromosomes. Figs. S4 shows characterization of the *caz-Xrp1* genetic interaction. Fig. S5 shows that heterozygosity for *Xrp1* mitigates gene expression dysregulation in *caz* mutant CNS. Table S1 lists human homologues of Xrp1. Tables S2, S3, and S4 list Xrp1^long^, Xrp1^Short^, and Caz-interacting proteins, respectively. Table S5 lists RNA-seq results. Table S6 lists human AT-hook genes. Table S7 lists oligonucleotide primers.

## Supplementary Material

Supplemental Materials (PDF)

Table S1 (PDF)

Table S6 (PDF)

Table S7 (PDF)

Table S2 (XLS)

Table S3 (XLS)

Table S4 (XLS)

Table S5 (XLS)
