# Supplementary material for: Xrp1 genetically interacts with the ALS-associated FUS orthologue caz and mediates its toxicity
Source: J Cell Biol. 2018 Nov 5;217(11):3947–64. doi: 10.1083/jcb.201802151 (PMC6219715; doi:10.1083/jcb.201802151)
Supplement: Supplemental Materials (PDF) [file JCB_201802151_sm.pdf]

Supplemental material

Mallik et al., https://doi.org/10.1083/jcb.201802151

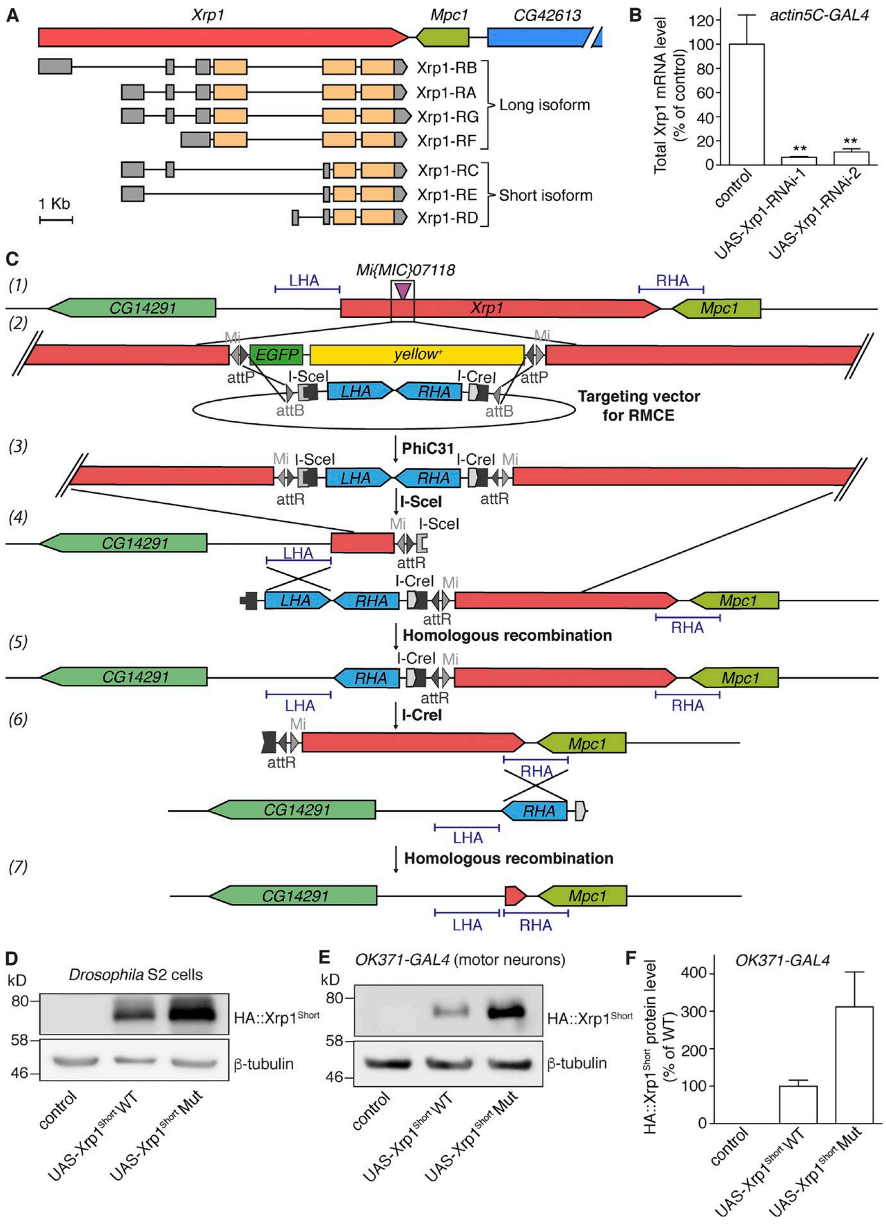

Figure S1. **Generation and characterization of *Xrp1* mutant and transgenic lines. (A)** Schematic representation of the Xrp1 genomic locus and the seven predicted Xrp1 transcripts (Xrp1-RA to Xrp1-RG) encoding either the short or long Xrp1 isoform. ORFs are colored orange; 5′ and 3′ UTRs are colored gray. **(B)** Efficiency of Xrp1 knockdown by transgenic RNAi expression. Real-time qPCR was used to quantify total Xrp1 transcript levels in the CNS of third instar larvae. Control animals (driver only) were compared with animals ubiquitously (*actin5C-GAL4*) expressing either of the two UAS-Xrp1-RNAi transgenes used in this study. Expression levels relative to control (100%) are shown. *n* = 8 (control), 5 (Xrp1-RNAi-1), and 7 (Xrp1-RNAi-2). **, P < 0.01; one-way ANOVA. Mean ± SEM. **(C)** Strategy used for generation of the $Xrp1^{KO}$ allele: (1) *Xrp1* genomic region with the insertion site of the *Mi{MIC}07118* transposable element indicated. The sequences used as left and right homology arms (LHAs and RHAs, respectively) are underlined. (2) Magnification of the *Mi{MIC}* element with flanking *Xrp1* sequences. The targeting vector used for recombination-mediated cassette exchange (RMCE) contains the left homology arm, preceded by an I-SceI restriction site, and the right homology arm, followed by an I-CreI restriction site, and flanked by attB recombination sites. (3) PhiC31 recombinase mediates recombination between the attP sites in the *Mi{MIC}* element and the attB sites in the targeting vector, resulting in exchange of the *Mi{MIC}* cassette by the targeting cassette. (4) Crossing with an I-SceI transgenic line results in a DNA double-strand break at the I-SceI site, and (5) in vivo homologous recombination between left homology arm sequences results in precise deletion of the *Xrp1* coding region left of the *Mi{MIC}* element. (6) Crossing with an I-CreI transgenic line results in a DNA double-strand break at the I-CreI site, and (7) in vivo homologous recombination between right homology arm sequences results in precise deletion of the *Xrp1* coding region right of the *Mi{MIC}* element. **(D–F)** The RGR-to-AAA mutation in the AT-hook motif does not reduce, but rather increases, the stability of the Xrp1 protein. **(D)** *Drosophila* S2 cells were cotransfected with a plasmid encoding *actin5C-GAL4* and plasmids encoding N-terminal HA-tagged Xrp1 (short isoform), either WT or AT-hook mutant (Mut). Cells transfected with the *actin5C-GAL4* plasmid alone were used as controls. Western blotting on protein extracts with antibodies against the HA-tag and β-tubulin (loading control) is shown. **(E)** WT or AT-hook mutant HA::Xrp1^{Short} was selectively expressed in motor neurons (*OK371-GAL4*) from UAS transgenes inserted in the same genomic landing site (VK31). Protein extracts from third instar larval CNS were used for Western blotting, using the same antibodies as in D. Driver-only animals were used as controls. **(F)** Quantification of HA::Xrp1^{Short} protein levels relative to β-tubulin in Western blots shown in E. Data are shown as percentages of UAS-Xrp1^{Short} WT. *n* = 5. P = 0.006 by one-way ANOVA.

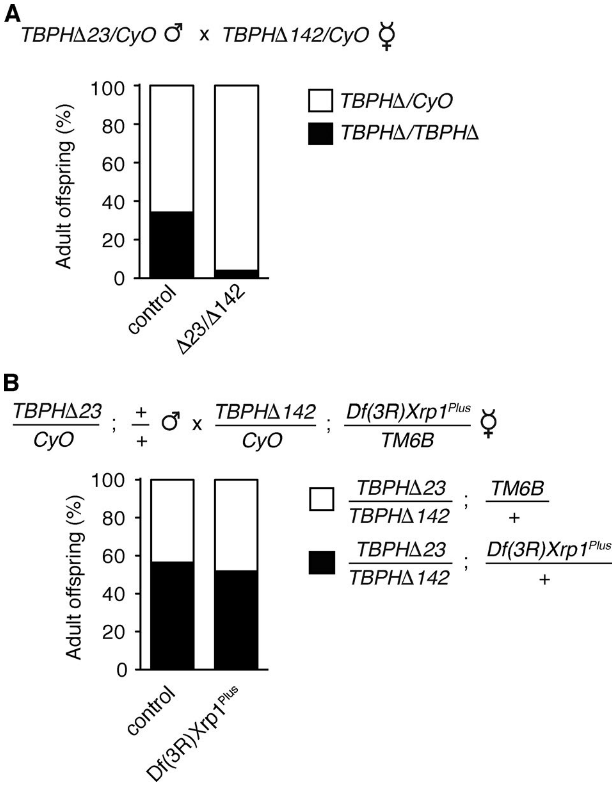

Figure S2. **Heterozygosity for *Xrp1* does not rescue the adult eclosion defect of *TBPH* mutant flies. (A)** Bar graph showing the relative frequency of adult F1 offspring for the indicated cross, using the *TBPHΔ23* and *TBPHΔ142* alleles. The offspring frequency of crosses between +/CyO males and females is shown as control. Since homozygous CyO flies die during development, the theoretically expected offspring frequency is 66.6% TBPHΔ/CyO and 33.3% TBPHΔ/TBPHΔ. The adult offspring frequency of TBPHΔ23/TBPHΔ142 flies is significantly reduced. **(B)** Relative frequency of adult non-CyO F1 offspring for the indicated cross. The theoretically expected offspring frequency is 50% TM6B/+ and 50% *Df(3R)Xrp1*^{Plus}/+. In control crosses, females carry a WT chromosome instead of *Df(3R)Xrp1*^{Plus}. Thus, heterozygosity for Xrp1 does not rescue *TBPH* mutant developmental lethality. Statistical analysis was performed using χ^2 test.

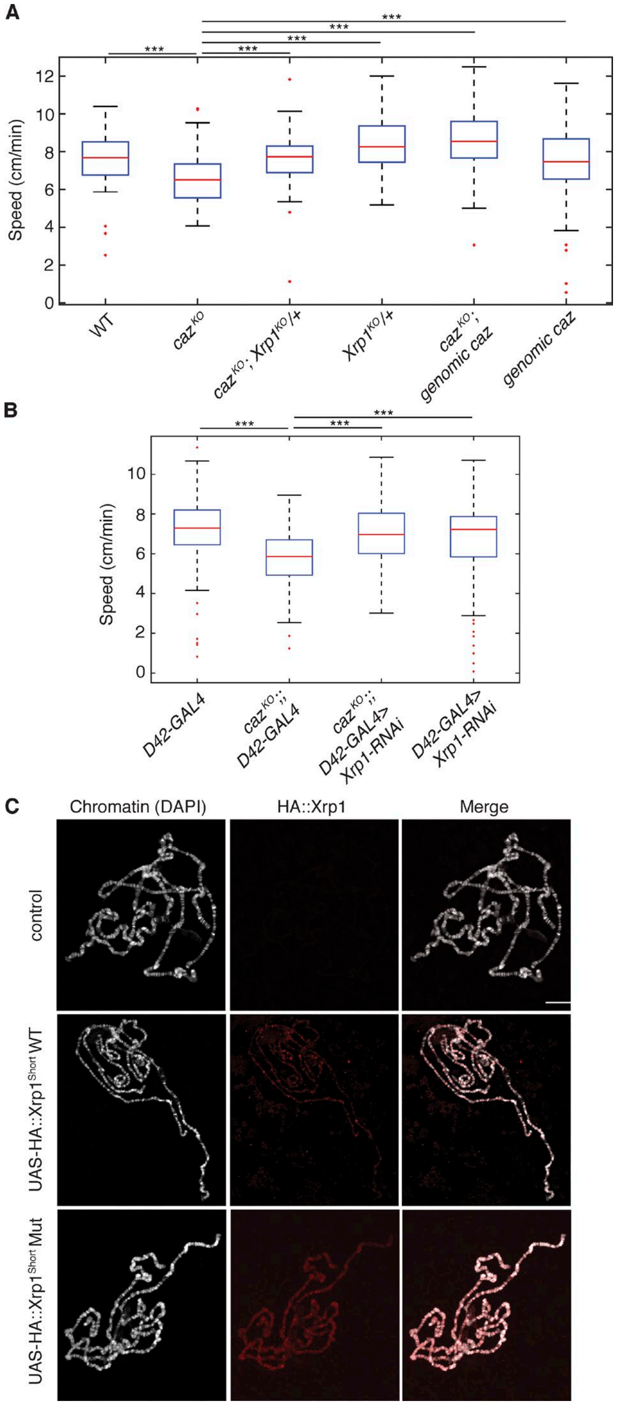

Figure S3. ***Caz*mutant larval motor defects are rescued by *Xrp1* heterozygosity or selective Xrp1 knockdown in motor neurons, and a subtle mutation in the Xrp1 AT-hook motif affects Xrp1 chromatin binding. (A)** Reduced crawling speed of *caz* mutant larvae is rescued by heterozygosity for *Xrp1* or a genomic *caz* transgene. n = 30–90. ***, $P < 0.0005$; one-way ANOVA. **(B)** Selective knockdown of Xrp1 in motor neurons (*D42-GAL4*) is sufficient to rescue the *caz* mutant locomotion defect. n = 90. ***, $P < 5 \times 10^{-7}$; one-way ANOVA. **(C)** A subtle mutation in the AT-hook motif alters the binding pattern of Xrp1 to polytene chromosomes. WT (middle) or AT-hook mutant (bottom) Xrp1^{Short} with N-terminal HA-tag was transgenically expressed in larval salivary glands. Anti-HA immunostaining revealed Xrp1 binding to polytene chromosomes, and DAPI was used to visualize banding patterns. Driver-only control (top) confirms specificity of the anti-HA antibody. Bar, 20 µm.

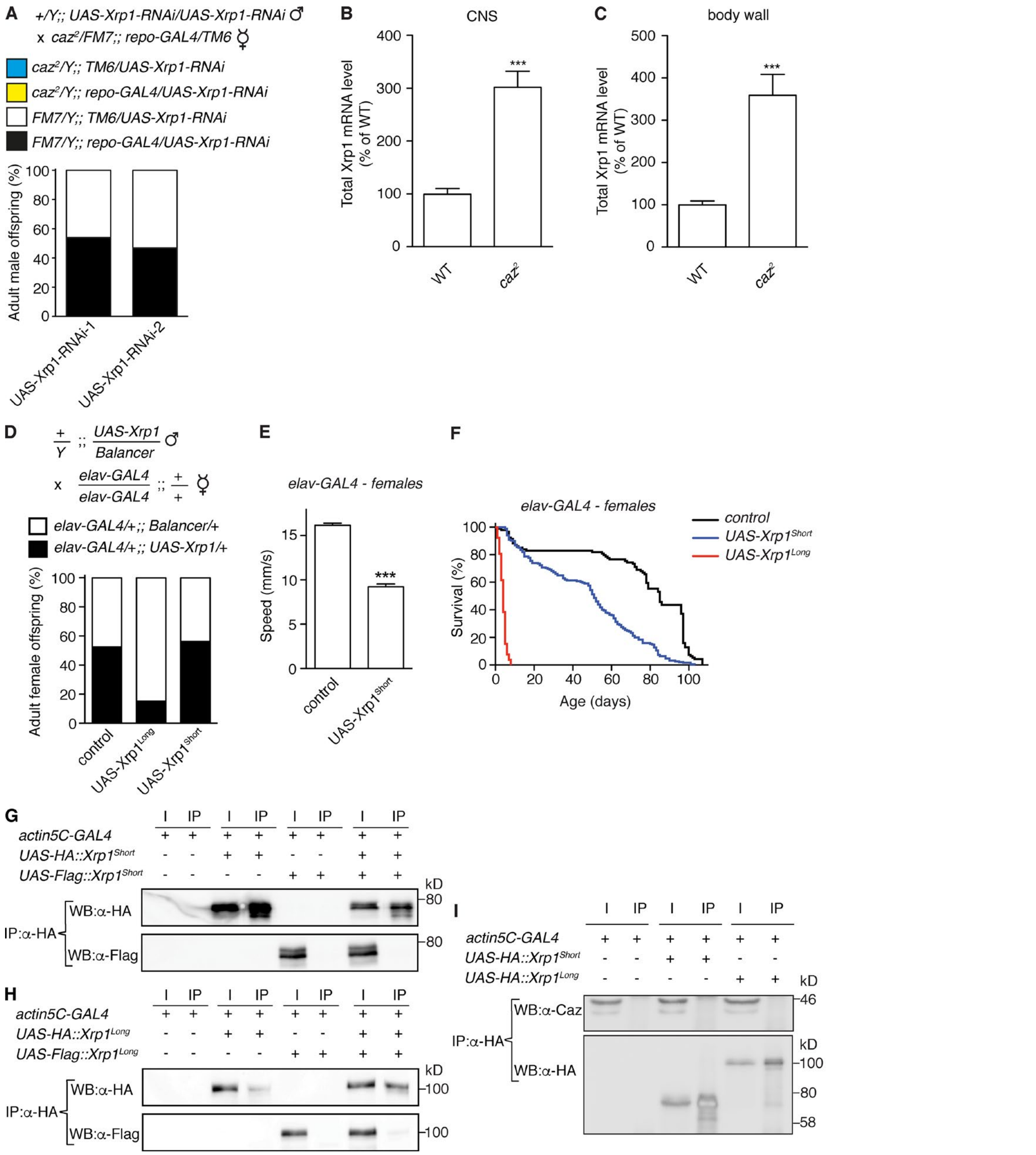

Figure S4. **Characterization of the *caz-Xrp1* genetic interaction. (A)** Selective knockdown of Xrp1 in glial cells does not rescue *caz* mutant pupal lethality. Relative frequency of adult male F1 offspring from the indicated cross is shown for two independent UAS-Xrp1-RNAi lines. No adult *caz*^{2} males were detected, even in the presence of the *repo-GAL4* driver to knock down Xrp1 selectively in glial cells. Note that a similar frequency of hemizygous FM7 males with or without panglial Xrp1 knockdown was found, showing that Xrp1 knockdown in glial cells does not induce developmental lethality. $n > 81$ per genotype. **(B and C)** Xrp1 expression is up-regulated in both CNS and body wall of *caz* mutant animals. Real-time qPCR was used to quantify total Xrp1 transcript levels in the CNS (B) and body wall (C) of third instar larvae, either WT or *caz*^{2}. Expression levels relative to WT (100%) are shown. $n = 8$. ***, $P < 0.001$; two-tailed unpaired *t* test. Mean ± SEM. **(D–F)** Selective neuronal Xrp1 overexpression phenocopies *caz* mutant phenotypes. **(D)** Relative frequency of adult female F1 offspring from the indicated cross that is heterozygous for either the balancer or UAS transgenes expressing the short or long Xrp1 isoforms. Neuron-selective (*elav-GAL4*) Xrp1^{Long} overexpression induces developmental lethality. $n > 144$ per genotype. **(E)** Average climbing speed of adult female flies selectively overexpressing Xrp1^{Short} in neurons (*elav-GAL4*) as compared with control (driver only) flies. $n > 100$ per genotype. ***, $P < 10^{-9}$; Mann-Whitney test. **(F)** Life span of female flies selectively overexpressing short or long Xrp1 isoforms in neurons (*elav-GAL4*) as compared with control (driver only) flies. $n = 26–189$ per genotype. **(G and H)** Coimmunoprecipitation experiments indicate that Xrp1 does not form homodimers. *Drosophila* S2 cells were transfected with a plasmid encoding *actin5C-GAL4* and different combinations of plasmids encoding N-terminal HA- or Flag-tagged Xrp1. Protein extracts were either directly used for Western blotting (WB) with anti-HA or anti-Flag antibodies (input [I] lanes) or immunoprecipitated with anti-HA antibody followed by Western blotting (immunoprecipitation [IP] lanes). Similar experiments were performed for the short (G) and long (H) Xrp1 isoforms. In both cases, immunoprecipitation of HA-tagged Xrp1 did not result in coimmunoprecipitation of Flag-tagged Xrp1. **(I)** Caz does not coimmunoprecipitate with Xrp1. *Drosophila* S2 cells were cotransfected with a plasmid encoding *actin5C-GAL4* and plasmids encoding N-terminal HA-tagged Xrp1, either the short or the long isoform. Cells transfected with the *actin5C-GAL4* plasmid alone were used as control. Protein extracts were prepared and used for immunoprecipitation with anti-HA antibodies. Western blotting of immunoprecipitates and 5% of original extracts (input) was performed using anti-HA or anti-Caz antibodies.

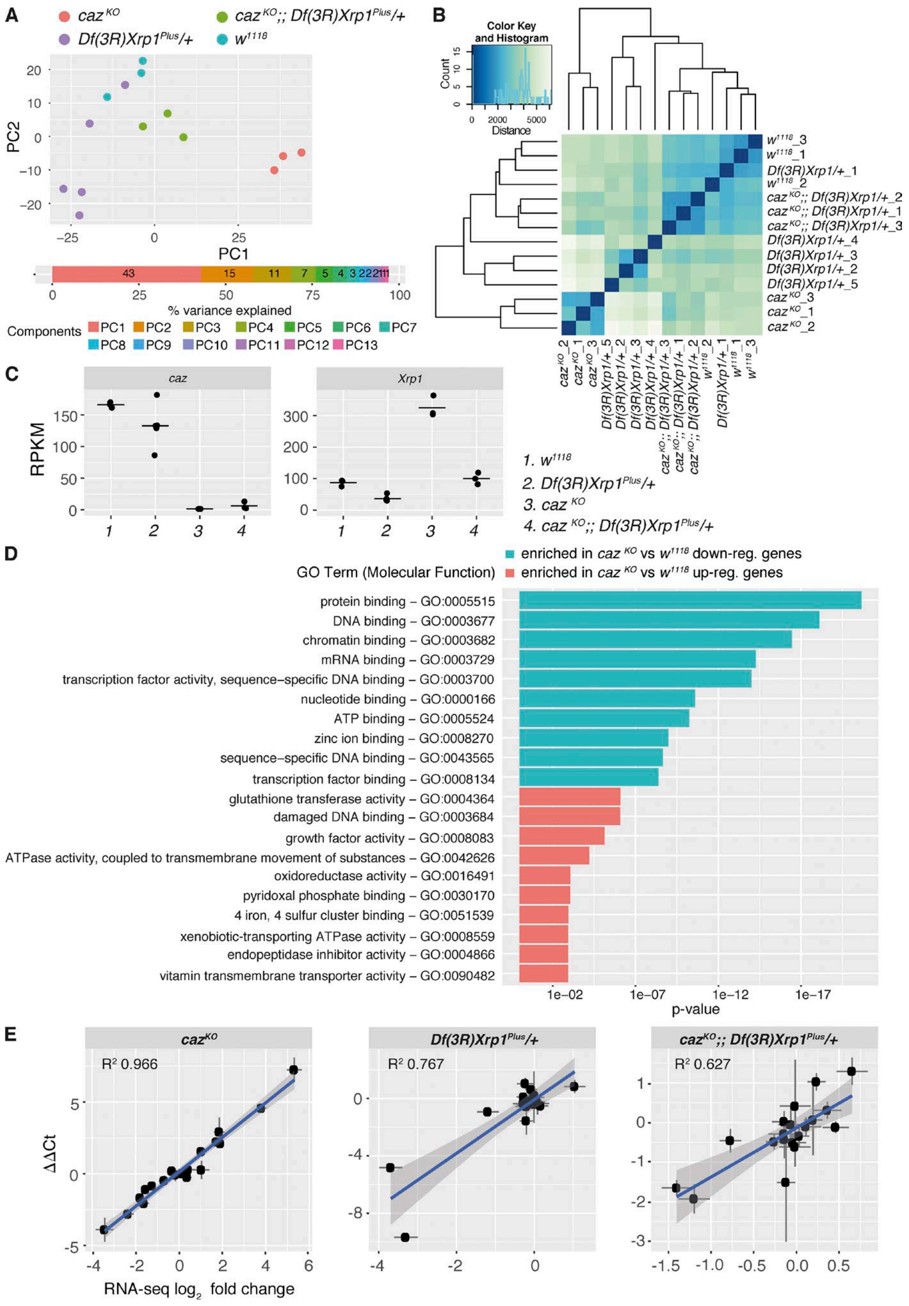

Figure S5. **Heterozygosity for *Xrp1* mitigates gene expression dysregulation in *caz* mutant CNS. (A)** Principal component analysis plot for all samples included in the RNA-seq analysis (red dots, *caz*^{KO}; purple dots, *Df(3R)Xrp1*^{Plus}/+; green dots, *caz*^{KO};;*Df(3R)Xrp1*/+; blue dots, *w*^{1118}). The principal component analysis procedure performs an orthogonal transformation on the data to convert the observed gene expression values in each sample into a set of values of variables linearly uncorrelated known as principal components (PCs). This transformation is defined so that the first principal component explains the largest possible variance. The plot displays the distribution of the samples according to the first two principal components, which overall explain 58% of the variance found in the data. The percentage of variance explained by the rest of principal components is displayed as a cumulative bar plot underneath the principal component analysis plot. **(B)** Unsupervised hierarchical clustering of all RNA-seq samples. Entries in the matrix are color-coded according to the Manhattan distance between samples. The dendrograms in the margins represent linkage distance between clusters. **(C)** Distribution of RPKM values for *caz* (left) and *Xpr1* (right) across all samples. RPKM values for each replicate are represented as solid dots. Horizontal lines indicate the mean value for each condition. **(D)** Top 10 enriched GO terms (Molecular Function ontology) in the *caz*^{KO} versus *w*^{1118} comparison for the set of up-regulated (red) and down-regulated genes (blue). **(E)** Correlation analysis of the $\log_2$ fold change values obtained from the RNA-seq analysis and qPCR-derived ΔΔCt values for a panel of 19 genes. Each plot displays the regression line (solid blue), 95% confidence interval (gray shadow), and R^2 values. Error bars indicate SEM.

Table S1 is a separate PDF showing human homologs of Xrp1.

Table S2 is a separate Excel file showing Xrp1^{Long}-interacting proteins ranked according to fold enrichment.

Table S3 is a separate Excel file showing Xrp1^{Short}-interacting proteins ranked according to fold enrichment.

Table S4 is a separate Excel file showing Caz-interacting proteins ranked according to fold enrichment.

Table S5 is a separate Excel file showing a list of RNA-seq results for each gene from the DESeq2 analysis.

Table S6 is a separate PDF showing human genes encoding AT-hook proteins, including gene name, Ensemble Gene ID, Ensemble Protein ID, and chromosomal location.

Table S7 is a separate PDF showing name, sequence, and purpose of oligonucleotide primers used in this study.
