# Supplementary material for: Xrp1 genetically interacts with the ALS-associated FUS orthologue caz and mediates its toxicity
Source: J Cell Biol. 2018 Nov 5;217(11):3947–64. doi: 10.1083/jcb.201802151 (PMC6219715; doi:10.1083/jcb.201802151)
Supplement: Table S1 (PDF) [file JCB_201802151_TableS1.pdf]

**TABLE S1: HUMAN HOMOLOGS OF XRP1**

| Protein ID | Protein name | E-value with XRP1 |
|---|---|---|
| XP_005258748.1 | protein fosB isoform X1 | 1.1E-10 |
| NP_004355.2 | CCAAT/enhancer-binding protein alpha isoform a | 1.5E-09 |
| XP_011523007.1 | hepatic leukemia factor isoform X2 | 8.3E-09 |
| XP_016868299.1 | cyclic AMP-responsive element-binding protein 5 isoform X5 | 0.000000011 |
| XP_016876460.1 | jun dimerization protein 2 isoform X1 | 0.000000038 |
| NP_005186.2 | CCAAT/enhancer-binding protein delta | 0.000000059 |
| NP_001181986.1 | DNA damage-inducible transcript 3 protein isoform 2 | 0.000000083 |
| NP_005243.1 | proto-oncogene c-Fos | 0.00000014 |
| XP_016881877.1 | D site-binding protein isoform X1 | 0.00000024 |
| NP_005429.1 | fos-related antigen 1 isoform 1 | 0.00000042 |
| NP_002117.1 | hepatic leukemia factor isoform 1 | 0.00000057 |
| NP_001254492.1 | cAMP-responsive element modulator isoform 24 | 0.00000093 |
| XP_016859226.1 | fos-related antigen 2 isoform X3 | 0.0000013 |
| NP_004895.2 | cyclic AMP-responsive element-binding protein 5 isoform beta | 0.0000019 |
| NP_877962.1 | cyclic AMP-dependent transcription factor ATF-4 | 0.0000023 |
| NP_001343.2 | D site-binding protein | 0.0000023 |
| NP_919047.2 | cyclic AMP-responsive element-binding protein 3-like protein 2 isoform 1 | 0.0000025 |
| NP_005244.1 | fos-related antigen 2 | 0.0000026 |
| XP_016883059.1 | nuclear factor interleukin-3-regulated protein-like | 0.0000037 |
| NP_002220.1 | transcription factor jun-B | 0.0000050 |
| NP_005375.2 | nuclear factor interleukin-3-regulated protein | 0.0000085 |
| NP_061134.1 | basic leucine zipper transcriptional factor ATF-like 3 | 0.000012 |
| XP_016871216.1 | cAMP-responsive element modulator isoform X21 | 0.000012 |
| NP_612465.3 | basic leucine zipper transcriptional factor ATF-like 2 isoform 1 | 0.000012 |
| NP_001073007.1 | X-box-binding protein 1 isoform XBP1(S) | 0.000015 |
| NP_001025458.1 | cyclic AMP-dependent transcription factor ATF-3 isoform 1 | 0.000020 |
| XP_005250598.1 | cyclic AMP-responsive element-binding protein 3-like protein 2 isoform X1 | 0.000021 |
| NP_036200.2 | cyclic AMP-dependent transcription factor ATF-5 | 0.000022 |
| XP_006718443.1 | cyclic AMP-responsive element-binding protein 3-like protein 1 isoform X1 | 0.000023 |
| NP_001243019.1 | cyclic AMP-dependent transcription factor ATF-2 isoform 1 | 0.000024 |
| NP_003207.1 | thyrotroph embryonic factor isoform 1 | 0.000025 |
| NP_443086.1 | cyclic AMP-responsive element-binding protein 3-like protein 1 | 0.000030 |
| NP_006390.1 | basic leucine zipper transcriptional factor ATF-like | 0.000031 |
| NP_001123532.1 | cyclic AMP-dependent transcription factor ATF-7 isoform 3 | 0.000034 |
| XP_016878722.1 | transcription factor Maf isoform X1 | 0.000060 |

| | | |
|---|---|---|
| NP_001273897.1 | transcription factor jun-D isoform deltaJunD | 0.000065 |
| NP_004372.3 | cyclic AMP-dependent transcription factor ATF-6 beta isoform a | 0.000078 |
| XP_016874825.1 | cyclic AMP-dependent transcription factor ATF-1 isoform X3 | 0.000081 |
| XP_016858890.1 | cyclic AMP-responsive element-binding protein 1 isoform X8 | 0.00015 |
| NP_002219.1 | transcription factor AP-1 | 0.00016 |
| NP_002219.1 | transcription factor AP-1 | 0.00016 |
| NP_005452.2 | transcription factor MafB | 0.00020 |
| NP_031374.2 | cyclic AMP-dependent transcription factor ATF-6 alpha | 0.00025 |
| NP_874386.1 | cAMP-responsive element modulator isoform 4 | 0.00029 |
| NP_001177.1 | transcription factor BACH1 | 0.00048 |
| XP_011521880.1 | transcription regulator protein MafG | 0.00048 |

Human homologs of Xrp1 (E-value < 0.001) ranked according to E-value. Homology detection was done with HHpred against the human proteome, using the default settings (three multiple-sequence alignment iterations with HHblits). Protein isoforms from the same gene were removed. All homologs are against the C-terminal, bZIP domain. None of the homologs contains an AT-hook motif.
