# Supplementary material for: Xrp1 genetically interacts with the ALS-associated FUS orthologue caz and mediates its toxicity
Source: J Cell Biol. 2018 Nov 5;217(11):3947–64. doi: 10.1083/jcb.201802151 (PMC6219715; doi:10.1083/jcb.201802151)
Supplement: Table S6 (PDF) [file JCB_201802151_TableS6.pdf]

TABLE S6: HUMAN GENES ENCODING AT-HOOK PROTEINS

Gene name	Ensembl Gene ID	Ensembl Protein ID	Chromosomal location	Upregulated in FUS mutant mice	FUS splice target	FUS RNA target	PMID references
AHCTF1	ENSG00000153207	ENSP00000355464	Chr 1 q44			Yes	24204307, 22081015
AHDC1	ENSG00000126705	ENSP00000247087	Chr 1 p36.11				
ASH1L	ENSG00000116539	ENSP00000357330	Chr 1 q22			Yes	24204307, 22081015
ELF3	ENSG00000163435	ENSP00000352673	Chr 1 q32.1	Yes			27368346
FAM171B	ENSG00000144369	ENSP00000304108	Chr 2 q32.1			Yes	22081015, 22427648
BOD1L1	ENSG00000038219	ENSP00000040738	Chr 4 p15.33				
HMGA1	ENSG00000137309	ENSP00000363230	Chr 6 p21.31			Yes	22081015
SCML4	ENSG00000146285	ENSP00000358018	Chr 6 q21		Yes		25907258
ZBTB24	ENSG00000112365	ENSP00000230122	Chr 6 q21				
KMT2C	ENSG00000055609	ENSP00000262189	Chr 7 q36.1				
KMT2A	ENSG00000118058	ENSP00000436786	Chr 11 q23.3				
PHF21A	ENSG00000135365	ENSP00000323152	Chr 11 p11.2			Yes	22081015
BAZ2A	ENSG00000076108	ENSP00000448760	Chr 12 q13.3		Yes	Yes	23023293, 22081015
HMGA2	ENSG00000149948	ENSP00000384026	Chr 12 q14.3			Yes	22081015
PDS5B	ENSG00000083642	ENSP00000313851	Chr 13 q13.1			Yes	24204307, 22081015
SRCAP	ENSG00000080603	ENSP00000262518	Chr 16 p11.2		Yes	Yes	25907258, 22081015
GLYR1	ENSG00000140632	ENSP00000390276	Chr 16 p13.3			Yes	22081015
CBX2	ENSG00000262762	ENSP00000460115	Chr 17 q25.3	Yes		Yes	23023293, 22081015
SETBP1	ENSG00000152217	ENSP00000282030	Chr 18 q12.3			Yes	22081015
DOT1L	ENSG00000104885	ENSP00000381657	Chr 19 p13.3	Yes		Yes	23023293, 24204307, 22081015
PRR12	ENSG00000126464	ENSP00000394510	Chr 19 q13.33	Yes		Yes	27368346, 22081015
ZNF524	ENSG00000171443	ENSP00000301073	Chr 19 q13.42				
ZNF653	ENSG00000161914	ENSP00000293771	Chr 19 p13.2				
KMT2B	ENSG00000272333	ENSP00000398837	Chr 19 q13.12	Yes			27368346
PHF20	ENSG00000025293	ENSP00000363124	Chr 20 q11.22			Yes	22081015
PATZ1	ENSG00000100105	ENSP00000266269	Chr 22 q12.2				
MECP2	ENSG00000169057	ENSP00000301948	Chr X q28			Yes	22081015, 23023293
