# Supplementary material for: Xrp1 genetically interacts with the ALS-associated FUS orthologue caz and mediates its toxicity
Source: J Cell Biol. 2018 Nov 5;217(11):3947–64. doi: 10.1083/jcb.201802151 (PMC6219715; doi:10.1083/jcb.201802151)
Supplement: Table S7 (PDF) [file JCB_201802151_TableS7.pdf]

TABLE S7: NAME, SEQUENCE, AND PURPOSE OF OLIGONUCLEOTIDE PRIMERS USED IN THIS STUDY

Primer name	Sequence (5' to 3')	Purpose
Xrp Del FW1	GCGCAGTTTCTTCCAGCGAC	Verification of *Xrp1*$^{Ex\text{-}long}$
Xrp Del FW2	TCAAGGCCAATCGCAAAGTG	
Xrp Del REV1	CGGCGCAACAAGTTAGAAGG	
Xrp Del REV2	ACACCTCTTAACGCTGCAAC	
CG14291 Del FW1	GTGCGTCCAGATTGGGAGTTTG	Verification of *Df(3R)Xrp1*^{Plus}
CG14291 Del FW2	CCGAGGTGAAGGCGTTGTTG	
CG42613 Del REV1	TGTACTTCGGAGCCCGTTCG	
CG42613 Del REV2	TTATACTCCTCGTCGGCAAC	
Xrp1 Long EagI FW	GCTACGGCCGATGATCCAGGAGCCAGC	Generation of Xrp1^{Long}
Xrp1 short NotI FW	GCTAGCGGCCGCATGTTTGCCGAGGAGGATC	Generation of Xrp1^{Short}
Xrp1 XhoI REV	GCTACTCGAGTCAGTCCTGCTCCTGCTTA	Generation of Xrp1^{Long} and Xrp1^{Short}
caz FW	CAACGACATGATCACCCAGG	Quantitation of caz mRNA
caz REV	CATTGGTGTCGTCGTAGGTG	
Xrp1 Sh Junc FW1	ATGATCGGTTCGAGGCTCC	Quantitation of total Xrp1 mRNA
Xrp1 Sh Junc REV1	ATCCTCTACGATGTCTGCATGG	
Xrp1 Long FW	TTGAAGAGATAGACGTTCCGGTG	Quantitation of Xrp1 Long mRNA
Xrp1 Long REV	AGATCCTCCTCGGCAAACATG	
rp49 FW	CCAGTCGGATCGATATGCTAA	Quantitation of rp49 control mRNA
rp49 REV	ACCGTTGGGGTTGGTGAG	
EifTuM FW	CATGTCCTTCATCCAACTGCA	Quantitation of EifTuM control mRNA
EifTuM REV	AATGAGCTTGGTGTCTTCGCC	
Xrp1_LHA_FW	AGTCAAGCTTCAAAACGTCGTGAGACAGTTTGAACAGCTGTT GCAACGTGTGG	Amplification of left homology arm for Xrp1 gene targeting
Xrp1_LHA_REV	AGTCGAATTCAGTATTGTTACTAACTGACTCAACTGCGAGC	
Xrp1_RHA_FW	AGTCGAATTCCGTAGCCATAGAATAGAAATTGCGGAGC	Amplification of right homology arm for Xrp1 gene targeting
Xrp1_RHA_Rev	AGTCGGTACCATTACCCTGTTATCCCTAGCAGCAGGCGTAAA TGTAATCCTTGC	
